# MLL/WDR5 complex recruits centriolar satellite protein Cep72 to regulate microtubule nucleation and spindle formation

**DOI:** 10.1126/sciadv.adn0086

**Published:** 2024-12-11

**Authors:** Swathi Chodisetty, Aditi Arora, Kausika Kumar Malik, Himanshu Goel, Shweta Tyagi

**Affiliations:** ^1^ Laboratory of Cell Cycle Regulation, Centre for DNA Fingerprinting and Diagnostics (CDFD), Hyderabad 500039, India.; ^2^ Graduate Studies, Manipal Academy of Higher Education, Manipal 567104, India.; ^3^ Graduate Studies, Regional Centre for Biotechnology, Faridabad 121001, India.; ^4^ Hunter Genetics, Hunter New England Local Health District (HNELHD), Waratah, NSW 2298, Australia.; ^5^ School of Medicine and Public Health, University of Newcastle, Callaghan, NSW 2308, Australia.

## Abstract

Dysfunction of the centrosome, the major microtubule-organizing center of the cell, is implicated in microcephaly. Haploinsufficiency of mixed-lineage leukemia (MLL/KMT2A) protein causes Wiedemann-Steiner syndrome (WSS), a neurodevelopmental disorder associated with microcephaly. However, whether MLL has a function at the centrosome is not clear. Here, we show that loss of the MLL/WDR5 complex affects microtubule nucleation and regrowth. MLL/WDR5 localize to the pericentriolar material and interact with centriolar satellite protein Cep72 and γ-tubulin ring complex proteins (γ-TuRCs). MLL/WDR5 promote the localization of γ-TuRCs and structural proteins like AKAP9 to the centrosome during interphase and mitosis, a phenotype also observed in cells derived from patients with WSS. During mitosis, loss of MLL, WDR5, and Cep72 affects spindle formation and leads to misaligned chromosomes. Last, we show that MLL and WDR5 recruit Cep72 to the centrosome. Our studies provide insight into an undiscovered role of MLL at the centrosome and elucidate how centriolar satellite proteins like Cep72 can be recruited to the centrosome.

## Teaser

MLL/WDR5 complex recruits centriolar satellite protein Cep72 to regulate microtubule nucleation at the centrosome.

## INTRODUCTION

Histone 3 lysine 4 (H3K4) histone methyltransferases (HMTs) are complex enzymes that are responsible for the genome-wide deposition of H3K4 me1/2/3 mark and required for the maintenance of chromatin in active state (*1*–*3*). In yeast, there is a single H3K4 HMT complex, whereas, in humans, there are six such complexes: mixed-lineage leukemia 1-4 (MLL1-4), SET Domain Containing 1A (SET1A), and SET1B (*4*–*6*). These proteins associate with four common structural components—WDR5, RbBP5, ASH2L and DPY30 (together known as WRAD)—to enhance their catalytic activity (*7*, *8*). It is difficult to define the essentiality of each H3K4 HMT complex in a function as they have both specific and redundant roles in cellular processes (*9*–*14*). Nonetheless, it is important to identify what functions these complexes participate in, so that we can gain a better understanding of their biology.

MLL1 or MLL [Lysine methyltransferase 2A (KMT2A)] is a large multi-domain protein that is proteolytically processed into MLL_N_ and MLL_C_ subunits, which associate to form a functional heterodimer (*15*, *16*). MLL_N_ consists of many domains, which target MLL protein to chromatin, whereas the MLL_C_ region constitutes of transcriptional activation domain (TAD) for transcriptional activity and Su(var) 3-9, enhancer-of-zeste, trithorax (SET) domain for its HMT activity. *mll*-null mice are embryonically lethal, but mouse models expressing MLL protein without SET domain are viable, indicating that MLL may have SET domain–independent functions (*9*, *11*, *12*). Mice heterozygous for *mll* are viable but show growth retardation (*9*), whereas conditional *mll*-knockout mice show impaired neurogenesis in the postnatal brain (*17*), indicating the crucial role of this protein in development. In humans, de novo heterozygous mutations in the *MLL* gene cause Wiedemann-Steiner syndrome (WSS), a neurodevelopmental disorder with distinctive facial features, indicating that haploinsufficiency of *MLL* is deleterious for growth. All affected individuals show growth retardation, psychomotor delay, and intellectual disability. About one-third of affected individuals have microcephaly (head circumference < 2 SD below the mean for age). *MLL* also undergoes frequent chromosomal translocations in leukemia where it fuses with ~120 different partner genes generating chimeric fusion proteins including proteins related to centrosomes or cytoskeletons (*18*). We have previously reported MLL on the centrosome and spindle apparatus, and loss of MLL gives rise to improper chromosome alignment, multipolar spindles, and spindle defects (*19*, *20*). Most of the microcephaly-related proteins localize to the centrosome and regulate cell division and cell fate. However, the role of MLL at this organelle is not clear.

Centrosomes act as the primary microtubule (MT)–organizing center (MTOC) in the cells and, by maintaining MT number and distribution, regulate cell shape, motility, intracellular transport, and bipolar spindle formation during cell division. Centrosomes contain two centrioles with surrounding pericentriolar material (PCM), whose dynamics affect the MT nucleation properties of the centrosome. Normally, MTs originate from either the centrosome or the Golgi apparatus, nuclear envelope, and MTs themselves (*21*). At the centrosomes, γ-tubulin and associated γ-tubulin complex proteins (GCPs) act as nucleating factors for the MTs by forming γ-tubulin ring complexes (γ-TuRCs) (*22*–*24*). γ-TuRC recruitment is mediated by multiple proteins of the centrosome such as pericentrin (PCNT), A-kinase anchoring protein 9 (AKAP9)/Centrosome and Golgi localized Protein Kinase N-associated protein(CG-NAP), ninein, and ninein-like proteins that have all been predicted to form a structural framework of centrosome (*25*, *26*). The function of centrosome structural proteins was majorly known to act as a scaffolding protein due to their size but advances in research showed multifunctionality. For example, AKAP9 has been shown to have a role in the cyclic adenosine 3′,5′-monophosphate (cAMP)/cAMP-dependent protein kinase signaling pathway, centriole duplication, and MT nucleation from both centrosomes and Golgi apparatus (*27*, *28*). AKAP9 has different regions for inhibiting or promoting MT nucleation when overexpressed (*29*). Recent studies show that these structural proteins are also regulated by the different cytoplasmic granules of proteins present around the centrosomes known as centriolar satellites (*30*, *31*). Centriolar satellite proteins like Cep72 target nucleating factors like γ-TuRCs to the centrosomes and ensure proper nucleation of MTs (*32*). Cep72 is also responsible for maintaining spindle pole stability and, hence, chromosome alignment during mitosis (*32*). It also affects centriole duplication by regulating the localization of CDK5 regulatory subunit associated protein 2 (CDK5RAP2) (*33*). However, how this “key” centriolar satellite protein is itself recruited to the centrosome is not yet understood.

Here, we undertook studies to understand whether MLL has a functional role at the centrosome. Our studies show that MLL and WDR5 regulate MT nucleation and regrowth by interacting with Cep72. MLL/WDR5/Cep72 form a complex with central proteins involved in MT nucleation like γ-tubulin and GCP2 and affect their levels at the centrosome during interphase and mitosis. While the centriolar satellite protein, Cep72, has been shown to recruit AKAP9 to centrosome (*32*), our studies here show that MLL/WDR5 recruit Cep72 to the centrosome. Further, B lymphocytes derived from patients with WSS also exhibit reduced levels of centrosome proteins and compromised MT nucleation. Our results assign a functional role to MLL at the centrosome and show how MLL participates in crucial functions like MT nucleation to promote genomic stability.

## RESULTS

### The MLL complex localizes to the centrosome

**Fig. 1.**
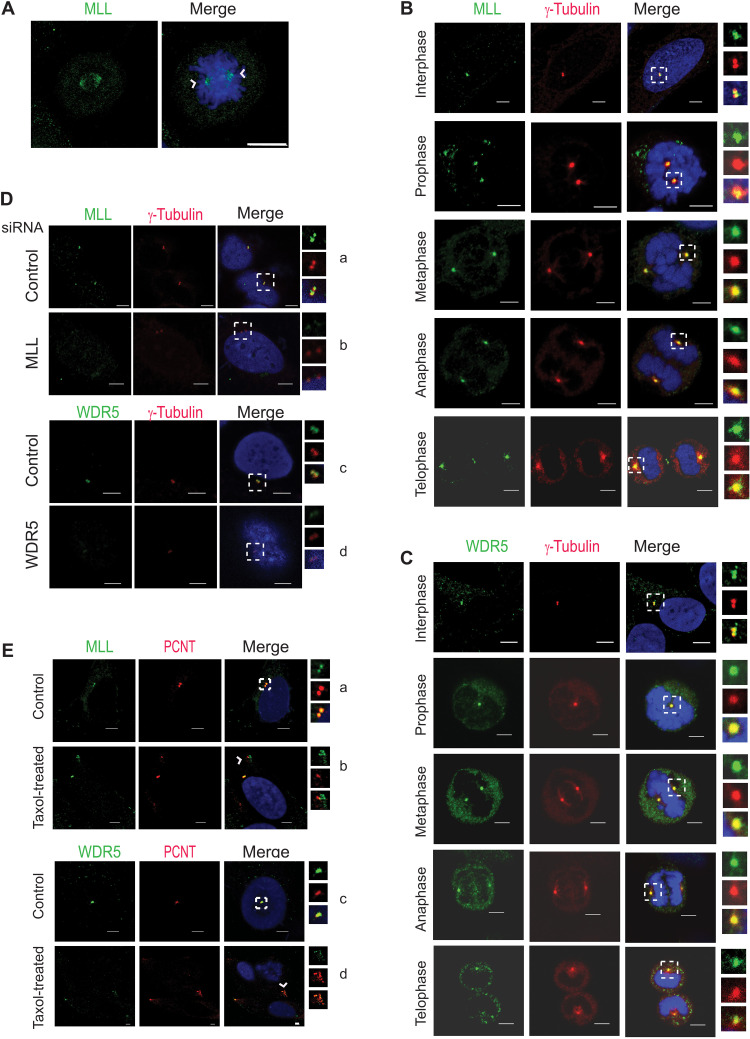
The MLL complex localizes to the centrosomes. (**A**) U-2OS cells were stained with α-MLL (green) to show the localization of MLL on the centrosomes and spindle apparatus in a cell undergoing mitosis. White arrowheads mark centrosomes; condensed chromosomes were stained with 4′,6-diamidino-2-phenylindole (DAPI; blue). (**B** and **C**) U-2OS cells were labeled with endogenous MLL_C_ (B) or WDR5 (C) (shown in green) and centrosomal marker γ-tubulin (red) using specific antibodies. DAPI is shown in blue. (**D**) Immunofluorescence staining (IFS) of U-2OS cells treated with control [(a) and (c)] or MLL (b) or WDR5 siRNA (d) is shown. The cells were stained with the respective protein antibodies (MLL or WDR5) to check for their specific localization on the centrosome. DAPI (blue) and γ-tubulin (red) staining are shown. (**E**) Distribution of MLL_C_ [green, (a) and (b)] or WDR5 [green, (c) and (d)] on the centrosomes was compared in Taxol-treated and untreated U-2OS cells. PCNT (red) was used as positive control. When quantified, 94.11% cells showed PCNT dissociation in Taxol-treated cell, while 88.88% showed MLL dissociation, and 81.13% showed WDR5 dissociation. [(A) to (E)] Scale bars, 5 μm. Zoomed-in images are shown in insets.

We have previously reported that MLL and WDR5 localize to spindle apparatus in mitosis and function as a complex (Fig.1A) (*19*). At this time, we also observed that MLL was localizing on mitotic chromatin, centrosome, central spindle, and midbody during various stages of mitosis (*20*). To determine whether MLL and its core-complex protein, WDR5, have a functional role at the centrosome, we undertook further studies here. We started by checking whether both these proteins localize to the centrosome in all stages of mitosis. Previously, we have optimized different fixation conditions to observe MLL localization on each mitotic structure (*20*). Here, we specifically used fixation conditions optimized to visualize the centrosome staining of MLL. The endogenous MLL, when stained with an antibody against the MLL_C_ subunit, showed robust co-localization with γ-tubulin in all stages of mitosis (Fig. 1B). MLL_N_ subunit could also be detected on the centrosome throughout mitosis with N-subunit specific antibody (fig. S1A). Similar to both subunits of MLL, we observed WDR5 staining on the centrosome in interphase as well as in mitosis (Fig. 1C). The staining of these endogenous proteins was specific as small interfering RNA (siRNA) knockdown of MLL (fig. S1B) or WDR5 (fig. S1C) reduced their staining on the centrosome (Fig. 1D). This was further quantified with PCNT as a reference (fig. S1, D and E). The PCNT signal did not show much change even when MLL and WDR5 were markedly reduced on the centrosome (fig. S1, D and E). We also checked for MLL and PCNT signals in wild-type (*mll1*^+/+^) mouse embryonic fibroblasts (MEFs; fig. S1F). The WT MEFs showed a clear MLL signal on the centrosome, which was significantly reduced in *mll*-null (*mll*^−/−^) but not the PCNT signal. Together, our data indicate that MLL and WDR5 show specific staining on the centrosome in various cells (*20*).

Given the role of MLL as an HMT and the fact that centrosomes are known to serve as a platform for transitioning signaling proteins, although we observed MLL and WDR5 on the centrosome throughout the cell cycle, we performed additional assays to reveal their centrosome-specific characteristics. Most spindle pole proteins require intact MTs to accumulate at the centrosomes, while integral components of centrosome are believed to localize to the centrosome in an MT-independent manner (*34*, *35*). Therefore, we subjected the cells to cold treatment to depolymerize the MTs (fig. S2A). Unexpectedly, we observed that MLL and WDR5 persisted on the centrosome, even after MT depolymerization, suggesting that the localization of the MLL complex was not dependent on the MTs [compare control to cold-treated samples in fig. S2 (A and B)]. We noticed that, in cold-treated samples, MLL exhibited many foci/condensates and all of them were not at the centrosome. Therefore, it is likely that cold treatment or depolymerization of MTs affects the distribution of MLL on other organelles/in other processes in the cytoplasm.

**Fig. 2.**
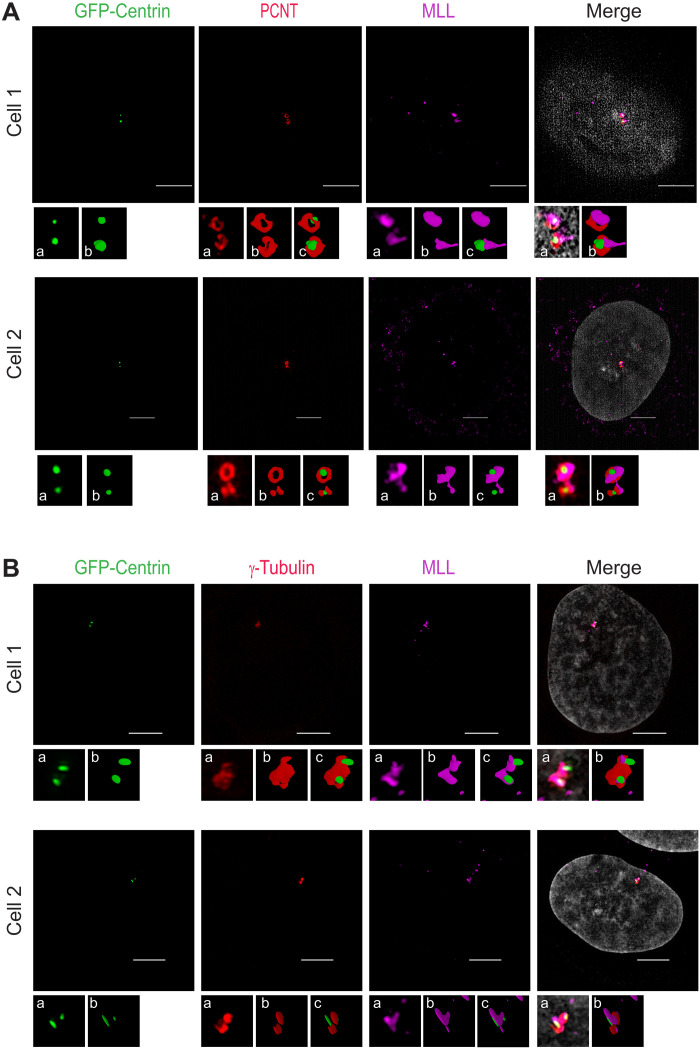
The MLL complex localizes to the centrosomes. (**A** and **B**) Structured illumination microscopy (SIM) images of U-2OS cells stably expressing GFP-Centrin (green), stained for MLL (magenta) and the centrosome markers, PCNT [red (A)] or γ-tubulin [red (B)], are shown. Zoomed inset (a) shows the multiple intensity projection, and insets (b) and (c) show the 3D full-resolution surface reconstruction of the above SIM images in respective channel or with Centrin, respectively. Scale bars, 5 μm.

In contrast to the cold treatment, Taxol treatment stabilizes the MT polymers. Prolonged treatment with Taxol induces loss of centrosome integrity, and PCNT along with PCM dissociates from centrosome (*36*). Upon Taxol treatment, we observed that both MLL and WDR5 dissociated from the centrosome just like the PCNT (Fig. 1E and fig. S2C), indicating that both MLL and WDR5 are associated with the PCM. To probe this result further, we used cells stably expressing green fluorescent protein (GFP)–Centrin to mark centrioles and stained these cells for PCNT to mark the PCM. Three-dimensional structured illumination microscopy (3D-SIM) revealed two centrioles and, as shown in the image in Fig. 2A, PCNT formed a toroid structure surrounding the centriole. We observed that, unlike most centrosome proteins, MLL did not form a torus and seem to lie at the proximal end of the centrioles. Further analysis of other images with PCNT and γ-tubulin (Fig. 2, A and B) revealed that MLL is closely associated with the centrioles, and it also connects them. In some images, it displayed protrusion, resembling the centrosomal linker proteins that generally show fibrous staining (although the linkers are not closely associated with the centrioles). It seems to extend to one side of the centriole forming a distinct structure. With its large structure and several coiled-coil domains, it is conceivable that MLL is a component of the structural framework of the centrosome. Together, our findings reveal that MLL, along with WDR5, is present on the centrosome associated with the PCM, throughout the cell cycle.

### The MLL complex has a role in MT nucleation and regrowth

**Fig. 3.**
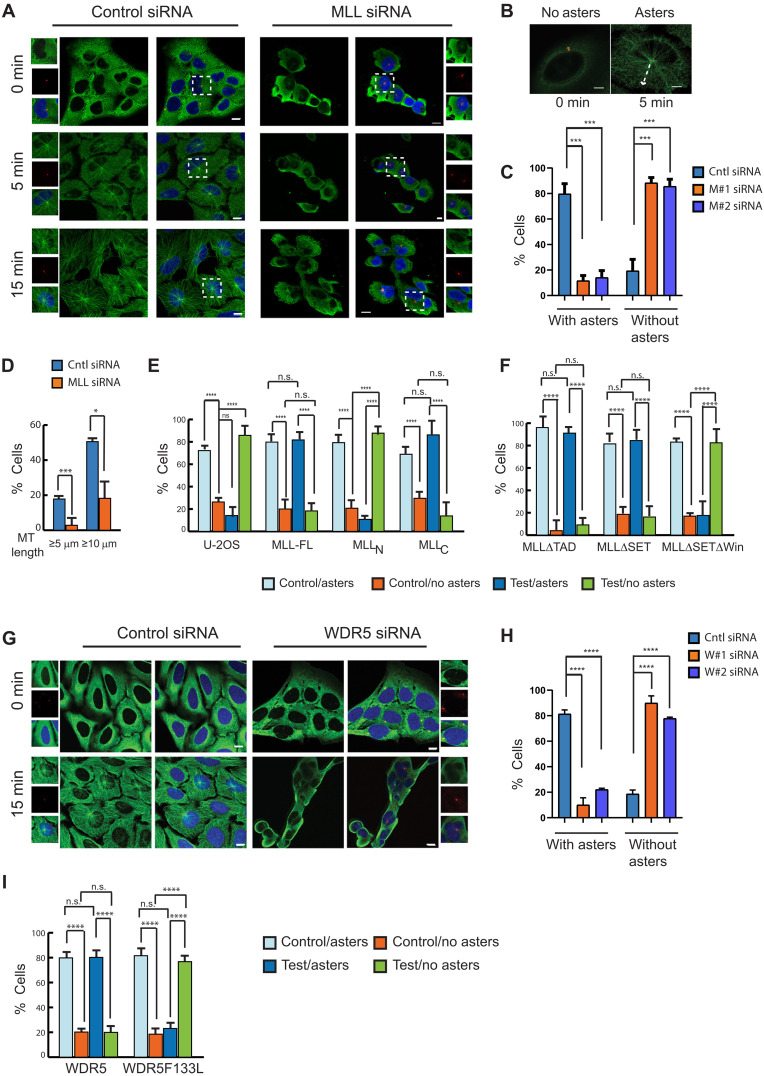
MLL and WDR5 regulate MT nucleation and regrowth. (**A**) Cells treated with control (left) or MLL siRNA#2 (right) were fixed after MT regrowth and stained with α-tubulin (green), PCNT (red), and DAPI (blue). (**B** to **D**) MT regrowth assay was performed, and cells were scored for presence or absence of asters after 5 min (B) or 15 min [(C) and (D)] of MT regrowth as shown. (C) U-2OS cells treated with two different MLL siRNA (M#1 and M#2). The percentage of cells was quantified and shown in the bar graph (*n* = 100, *m* = 2 experiments). ***P ≤ 0.0008 [two-way analysis of variance (ANOVA) with Tukey’s multiple comparisons test]. (D) Length of MT was measured (in micrometers) in control and MLL siRNA-treated samples. *P ≤ 0.01 and ***P ≤ 0.0006 (unpaired Student’s *t* test). [(C) and (D)] Error bars represent SEM. n.s., not significant. (**E** and **F**) MT regrowth assay in U-2OS cells stably expressing (E) MLL full length (MLL-FL), MLL_N_ subunit, and MLL_C_ subunit as well as (F) MLLΔTAD, MLLΔSET, and MLL ΔSETΔWIN after treatment with control or MLL#2 siRNA is shown. Cells with and without asters were scored (*n* = 200 cells, *m* = 2 experiments) for each cell line. Error bars represent SEM. ****P ≤ 0.0001 (two-way ANOVA with Tukey’s multiple comparisons test). (**G**) U-2OS cells treated with control or WDR5 siRNA#2 were stained with α-tubulin (green), PCNT (red), and DAPI (blue). Cells were observed for MT regrowth at the indicated time points after the cold treatment. [(A), (B), and (G)] Scale bars, 10 μm. Zoomed-in images are shown in insets. (**H** and **I**) Cells with and without asters are quantified after 15 min MT regrowth in (H) control, WDR5 siRNA (W#1 and W#2)–treated U-2OS cells, or (I) in cell lines expressing exogenous siRNA-resistant WDR5 or WDR5 F133L (*38*), after treating with Control or WDR5 siRNA#2 and represented (*n* = 100, *m* = 2 experiments). Error bars represent SEM. ****P ≤ 0.0001 (two-way ANOVA with Tukey’s multiple comparisons test).

We investigated the function of MLL in MT nucleation by carrying out MT regrowth assays (*37*). After treating the cell with control or MLL siRNA, the MTs were depolymerized by cold treatment. As shown in Fig. 3A, all the cells showed depolymerized MTs at the start of the experiment (0 min). The cells were then incubated at 37°C for “recovery.” Control cells showed MT nucleation from the centrosome very rapidly (Fig. 3, A and B). In contrast, MLL-depleted cells were significantly delayed in their MT nucleation. At this time MTs could be seen nucleating from centrosomes as a radial display called “aster” in control cells (see Fig. 3B). To give enough time to MLL siRNA-treated cells to recover, we choose to analyze cells at 15 min after incubation. A large number of control cells displayed aster formation 5 min after recovery, and, by 15 min, more than 80% cells had fully recovered, with long MTs reaching the cell cortex (Fig. 3, A and C). In contrast, very few cells showed aster formation in MLL-depleted samples even after 15 min. The same observations were made with a different MLL siRNA (siRNA#2, fig. S1B and Fig. 3C). MTs nucleated from centrosomal as well as from non-centrosomal MTOCs (*21*). To understand whether MLL affected the MT nucleation from the centrosome, we checked for aster formation after 25 min (fig. S3, B and C). We observed attenuated recovery of MTs globally, and this was especially notable at the centrosome in MLL or WDR5 siRNA-treated cells. When quantified, the number of cells lacking MT regrowth, particularly from the centrosome, was high in MLL or WDR5-depleted cell (fig. S3, C and D). When measured, about 50% of control cells had MT longer than 10 μm, while 20% displayed >5-μm MT length. Such MT length was rarely seen in MLL siRNA-treated samples, with barely 20% cells showing MT < 10 μm (Fig. 3D).

To understand how MLL is involved in MT nucleation, we decided to investigate which domain of MLL participated in this function. We performed complementation assays using stable cell lines expressing recombinant FLAG-tagged wild-type or mutant MLL protein as described previously (fig. S3E) (*38*). Using siRNA against 3′ untranslated region of MLL endogenous transcript (siRNA#2), which affected endogenous MLL transcript but not the transcript synthesized from the recombinant FLAG-tagged constructs, we were able to demonstrate that full-length and the C subunit of MLL, but not the N subunit, were able to restore aster formation like control siRNA-treated cells (Fig. 3E).

WDR5 interacts with MLL via its WDR5-interacting (WIN) motif (*39*). Consistent with our previous observations, where WIN motif of MLL and not the SET domain or TAD had a role in chromosome alignment (*19*), here, also, mutation in WIN motif of MLL could not rescue aster formation (Fig. 3F and fig. S3E). Our observations indicated that the interaction between MLL and WDR5 is essential for the MT nucleation activity of MLL. To gauge the participation of WDR5 in this process, we performed regrowth assay on WDR5 siRNA-treated samples and scored for cells with aster formation. Similar to MLL depletion, loss of WDR5 with two different siRNAs, attenuated the ability of cells to nucleate MTs significantly (Fig. 3, G and H, and fig. S3, B and D). This observation was recapitulated in complementation assays performed with cells stably expressing WDR5 F133L, a mutation that abrogates WIN motif-mediated binding (*38*, *40*), whereas wild-type WDR5 could rescue aster formation (Fig. 3I). The inability of MLL or WDR5 mutants to aid MT nucleation was not dependent on their ability to localize to the centrosome (fig. S3, F and G) (*20*).

To ascertain that this observation was not limited to U-2OS cells, we performed our MT nucleation assay in the non-transformed cell line IMR90-tert. Like in U-2OS cells, control siRNA-treated cells formed well-defined asters but the aster formation was visibly reduced in MLL and WDR5 siRNA-treated cells (fig. S3H), indicating that MLL/WDR5 participated in MT nucleation in both the transformed and the non-transformed cells. To sum up, our results indicate that the MLL/WDR5 complex may regulate MT nucleation and that the C subunit of MLL is the effector for this function.

Our results revealed that the WIN motif in MLL_C_ was required for the function of MLL in MT nucleation. To test which part of MLL_C_ was involved in its localization to the centrosome, we made deletions of MLL_C_ tagged with GFP at the N terminus (fig. S4A). Consistent with our results where the WIN motif of MLL has a role in MT nucleation (Fig. 3F), only cells expressing MLL_C_D3 (which harbors the WIN motif) were able to show MT nucleation as seen by aster formation (fig. S4, B and C). In addition, MLL_C_D3 was able to localize to the centrosome (fig. S4D) but not D1 or D2. To test whether the WIN motif had a role in the centrosome localization of MLL_C_, we made a mutation in the WIN motif (R3765A) in MLL_C_D3 (MLL_C_D3ΔWIN). Our results show that MLL_C_D3ΔWIN could localize to the centrosome (fig. S4, E and F), indicating that WIN motif of MLL and, hence, interaction with WDR5 were not required for MLL’s localization to the centrosome. Together, our results revealed that MLL_C_D3 was sufficient for localization and function of MLL in MT nucleation.

### The MLL/WDR5 complex interacts with multiple proteins involved in MT nucleation

To find out more about the role of MLL/WDR5 at the centrosome, we decided to look at their interacting partners. We have previously shown that Kif2A interacts with MLL/WDR5 complex (*19*). As Kif2A localizes to spindle poles and is involved in maintaining spindle dynamics, we interrogated its role with MLL/WDR5 in MT nucleation further (*41*). To that end, we performed immunofluorescence of Kif2A with γ-tubulin, and both proteins could be detected at the centrosome in interphase and mitosis (fig. S5A). The Kif2A staining diminished upon treatment with Kif2A siRNA (fig. S5, A and B). Next, we performed MT regrowth on the cells treated with Kif2A siRNA and observed that these cells displayed aster formation just like control cells (fig. S5, B to D). Hence, our results indicated that Kif2A is not involved with MLL/WDR5 in the process of MT nucleation. Next, we compared our phenotype to other motor proteins/interacting partners of WDR5 (*19*, *42*, *43*) and discovered that Cep72, an interacting partner of WDR5, shows the same phenotype as reported here (*32*). Cep72, a centriolar satellite protein, is suggested to be the central protein required for MT nucleation activity and maintaining the structural integrity of the centrosome (*32*). Cep72 was reported to be essential for the recruitment of proteins like AKAP9 and γ-TuRCs to the centrosome for MT nucleation (*32*). It is also required for centriole duplication by targeting microcephaly-associated proteins like CDK5RAP2 to the centrosome (*33*).

**Fig. 4.**
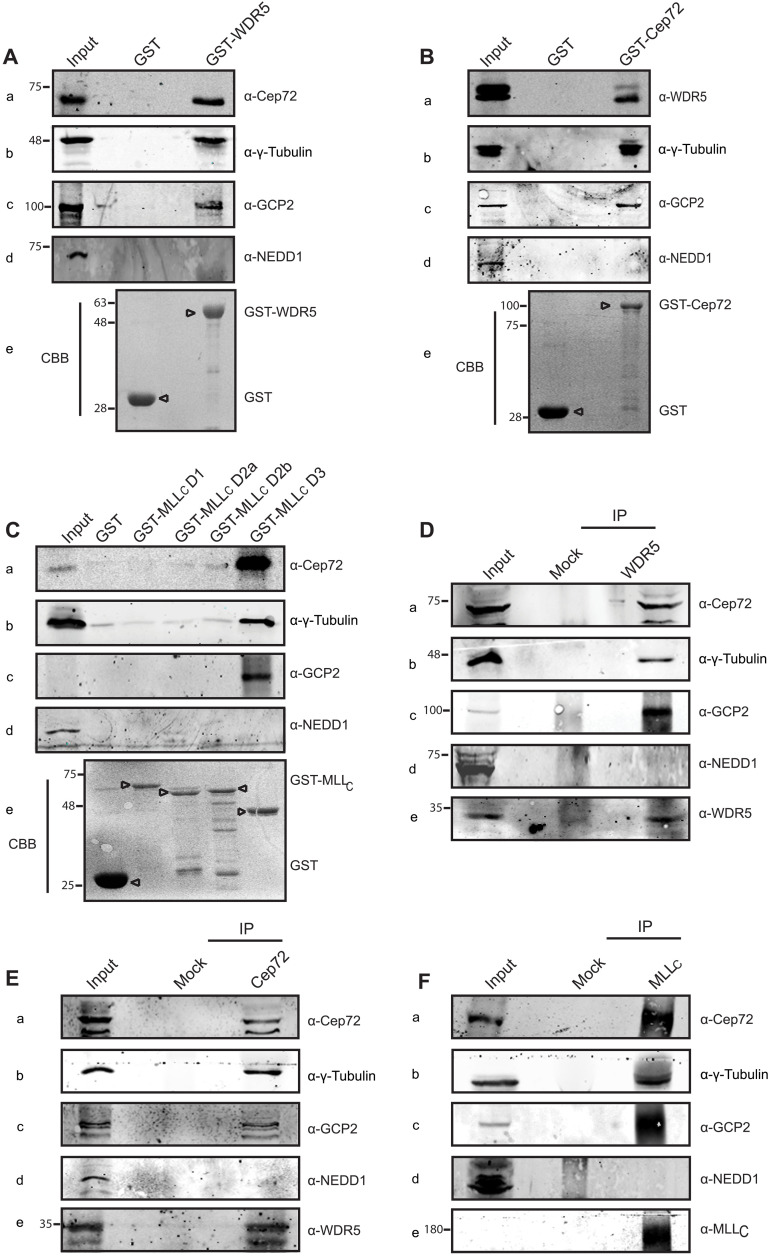
The MLL/WDR5 complex interacts with multiple proteins involved in MT nucleation. (**A**) HeLa cell lysates were subjected to affinity pull-down using glutathione agarose beads bound to glutathione *S*-transferase (GST) and GST-WDR5 proteins. Interactions were analyzed by SDS-PAGE followed by Western blot. Immunoblots were probed with anti-Cep72 (a), anti–γ-tubulin (b), anti-GCP2 (c), and anti-NEDD1 (d) antibodies as indicated. The amount of bead-bound GST or GST-WDR5 protein (arrowheads) used for the pull-down is shown by Coomassie brilliant blue (CBB) staining (e). Numbers on the left indicate the position of molecular weight markers (in kilodaltons). (**B**) Bacterially expressed GST and GST-Cep72 proteins were purified by glutathione agarose beads and used for affinity pull-down from HeLa cell lysate as described in (A). (**C**) MLL fragments were bacterially expressed as N-terminal GST fusions proteins (GST-MLL_C_D1, GST-MLL_C_D2a, GST-MLL_C_D2b, and GST-MLL_C_D3; see also fig. S4A). GST-MLL fragments were used for affinity pull-down from HeLa cell lysate. Exposure had to be decreased for blots in (a) and (c) to avoid oversaturation in pull-down lane. Immunoblots were probed with antibodies as indicated. The amount of bead-bound protein used in the pull-down is shown by CBB staining in the bottom panel. (**D** to **F**) Endogenous proteins were immunoprecipitated (IP) with specific antibodies against endogenous WDR5 (D), Cep72 (E), or MLL_C_ (F) and as indicated. Anti–immunoglobulin G antibody was used for mock IP. Immunoblots were probed with antibodies against specific centrosomal proteins as indicated on the right. Short exposures are shown for γ-tubulin (b) blot to avoid the signal from immunoglobulin heavy chain.

Consistent with previous reports, Cep72-GFP as well as endogenous Cep72 showed multiple foci around the centrosome resembling centriolar satellites (fig. S5, E and F) (*32*, *33*). We confirmed that the loss of Cep72 by RNA interference (RNAi) results in loss of signal of endogenous protein at the centrosome and attenuated MT nucleation (fig. S5, F to I). In light of above results, it seemed likely that Cep72 may be interacting with WDR5 to bring about the process of MT nucleation. To confirm that Cep72 interacts with WDR5, we performed an affinity protein interaction study with glutathione *S*-transferase (GST)–tagged WDR5. Pull-down experiments using GST or GST-WDR5 proteins showed that Cep72 interacted with WDR5 (Fig. 4A). We also probed for γ-tubulin, a well-known MT-nucleating protein; GCP2, a core subunit of γ-TuRC; and NEDD1 [gamma-tubulin ring complex targeting factor/gamma-tubulin targeting factor (GCP-WD)], a γ-TuRC–targeting protein (*23*, *44*). We tested WDR5 for interaction with γ-tubulin, GCP2, and NEDD1. While γ-tubulin and GCP2 showed interaction with WDR5, we could not detect NEDD1 in our pull-down, indicating that the WDR5 interacted with some of the proteins involved in MT nucleation (Fig. 4A). Similarly, we performed pull-down experiments with GST-Cep72 (Fig. 4B) and observed that WDR5 as well as γ-tubulin and GCP2 interacted with Cep72 but not NEDD1. We also used GST fusions of MLL_C_ deletions to interrogate which domain of MLL interacted with Cep72. From our earlier experience, we found MLL_C_ deletions highly unstable and difficult to express (*19*). Therefore, we divided the MLL_C_ protein into four parts (instead of three) such that D1 and D2 gave rise to three deletions and D3 remained as before (fig. S4A). We observed a robust interaction of MLL_C_ D3 with Cep72 and GCP2 (Fig. 4C). MLL_C_D3 also showed interaction with γ-tubulin but not NEDD1. Next, we immunoprecipitated endogenous WDR5, Cep72, and MLL_C_ using specific antibodies (Fig. 4, D to F) and observed that both WDR5 and MLL were able to pull down Cep72 and vice versa. Further, all the three proteins interacted with γ-tubulin and GCP2, suggesting that MLL/WDR5/Cep72 are part of a large protein complex involved in MT nucleation.

### Down-regulation of MLL/WDR5 affects the localization of centrosomal components during interphase and mitosis

**Fig. 5.**
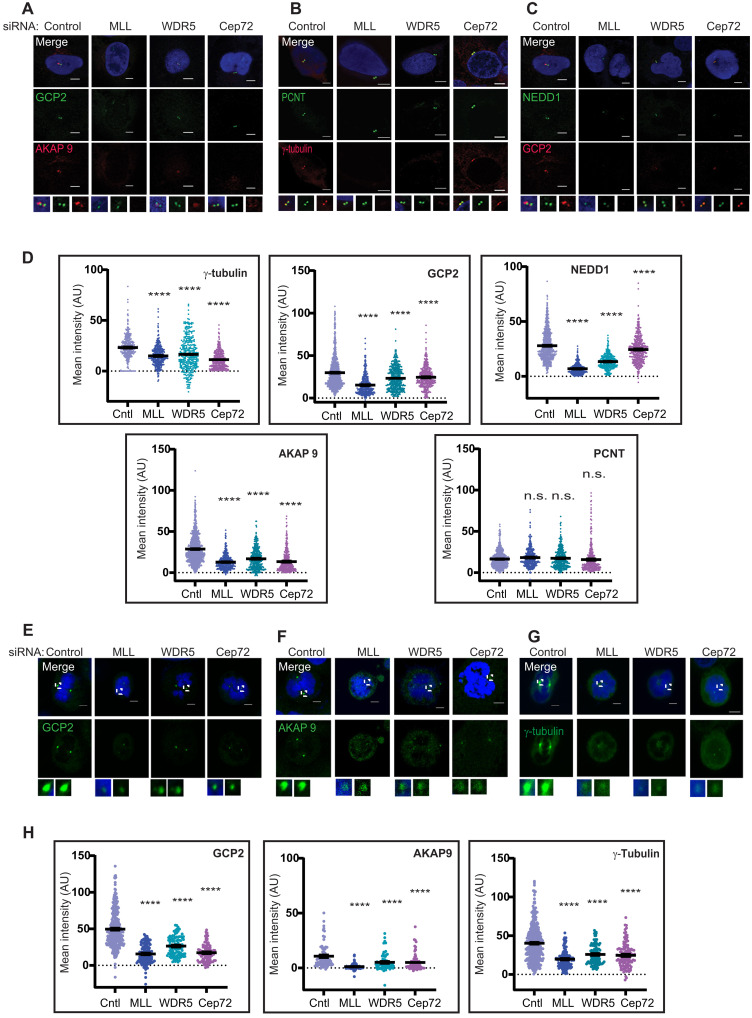
Down-regulation of MLL/WDR5 affects the localization of centrosomal components during interphase and mitosis. (**A** to **C**) U-2OS interphase cells were labeled with GCP2 [(A) green and (C) red], AKAP9 [(A) red], PCNT [(B) green], γ-tubulin [(B) red], and NEDD1 [(C) green] after treatment with control, MLL, WDR5, or Cep72 siRNA. Scale bars, 5 μm. See insets for magnified images. (**D**) Centrosome levels of γ-tubulin, GCP2, NEDD1, AKAP9, and PCNT were quantified in control, MLL, WDR5, or Cep72 siRNA-treated cells using Zen software, and mean intensities are shown as dot plot (*n* = 200, *m* = 2 experiments). Error bars represent SEM. ****P ≤ 0.0001 (one-way ANOVA with Bonferroni’s multiple comparisons test). (**E** to **G**) IFS of mitotic U-2OS cells was performed, and the cells were stained for either GCP2 (E), AKAP9 (F), or γ-tubulin (G) after 72 hours of treatment with control, MLL, WDR5, or Cep72 siRNA. Scale bars, 5 μm. (**H**) Centrosome levels of GCP2, AKAP9, and γ-tubulin were quantified in mitotic cells using Zen software. Mean intensities are shown (*n* = 50 centrosomes, *m* = 2 experiments). Error bars represent SEM. ****P ≤ 0.0001 (one-way ANOVA with Bonferroni’s multiple comparisons test). AU, arbitrary units.

Cep72 has been shown to target γ-TuRCs and AKAP9 to the centrosome to promote MT-nucleating activity (*32*). To explore whether MLL/WDR5 have a role in this pathway, we depleted MLL or WDR5 by siRNA. To assess the impact of Cep72 on the MT nucleation pathway and to make a comparison with the effects of MLL and WDR5, we also depleted Cep72. Consistent with previous report, Cep72 knockdown showed a decrease in the levels of γ-tubulin and GCP2 (*32*). We noticed a significant reduction in GCP2 and γ-tubulin upon MLL or WDR5 knockdown as well (Fig. 5, A to D). Depletion of MLL, WDR5, and Cep72 affected NEDD1 localization on the centrosome although NEDD1 did not show interaction with any of the three proteins (Fig. 5, C and D). Of the two large structural coiled coil proteins, which anchor γ-TuRCs, Cep72 affects the recruitment of AKAP9 but not PCNT (*32*). Consistent with our hypothesis that MLL/WDR5/Cep72 act together in this pathway, the levels of AKAP9 were reduced significantly in MLL/WDR5 siRNA-treated cells (Fig. 5, A, B, and D). In direct contrast to AKAP9, the levels of PCNT remained largely unchanged. Our RNA-sequencing (RNA-seq) experiments did not reveal differential regulation of the transcript of any centrosome proteins, including those being considered here (*45*). Consistent with the RNA-seq results, when checked by Western blot, γ-tubulin, GCP2, and NEDD1 proteins showed similar levels in control and test siRNA-treated samples (fig. S6, A and B).

Our results, so far, show that MLL/WDR5 recruit γ-TuRCs and AKAP9 to the centrosome during interphase to promote MT nucleation. To find out whether MLL/WDR5/Cep72 carry out a similar role during mitosis, we tested whether the levels of structural and nucleating proteins are affected at the centrosome upon their knockdown in cells synchronized in mitosis. We noticed an increase in the level of centrosome proteins during mitosis (Fig. 5, compare A to C with E to G), probably due to PCM expansion. Nonetheless, loss of MLL, WDR5, or Cep72 affected the levels of GCP2, γ-tubulin, NEDD1, and AKAP9 (Fig. 5, E to H, and fig. S6C). Unexpectedly, in contrast to the previous report (*32*), we detected a substantial decrease in PCNT levels upon loss of MLL, WDR5, or Cep72 in mitotic cells (fig. S6D). As PCNT is recruited through the dynein and centriolar satellite protein–mediated transport (*46*) and we observed change in levels of PCNT only in mitosis, decreased levels of AKAP9 may likely disturb the process of PCM expansion and affect the recruitment of other structural components like PCNT to the centrosome. To sum up, MLL, WDR5, and Cep72 target γ-TuRCs, NEDD1, and AKAP9 to centrosome in interphase and mitosis.

### Loss of MLL/WDR5 affects MT nucleation and spindle formation during mitosis

**Fig. 6.**
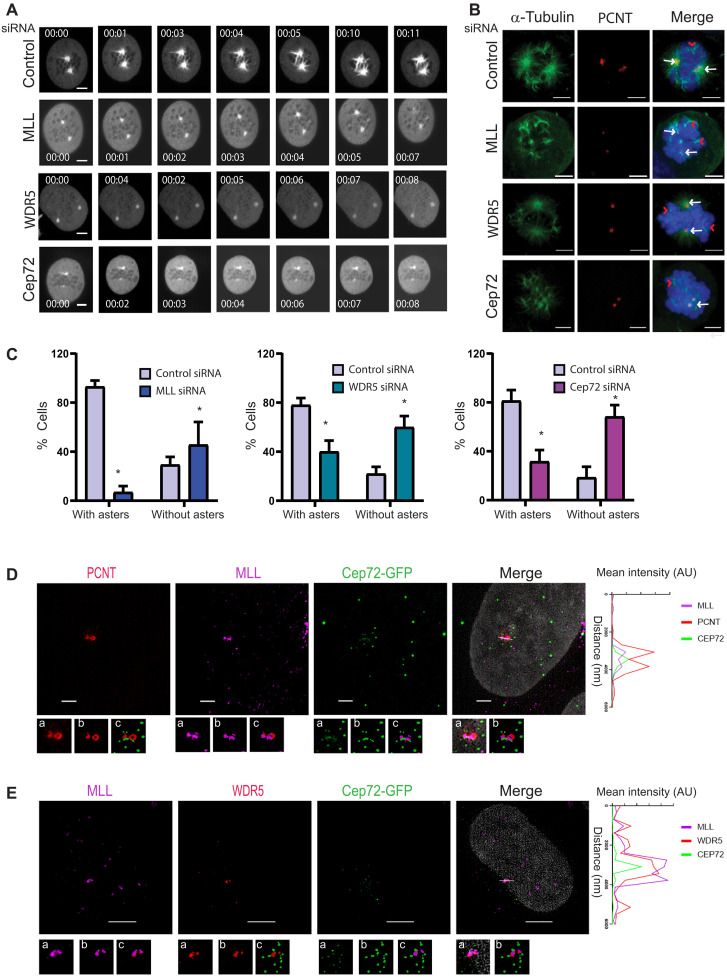
Loss of MLL/WDR5 affects MT nucleation and spindle formation during mitosis. (**A**) U-2OS cells stably expressing GFP-tagged tubulin and mCherry-tagged histone 2B were imaged in mitosis using time-lapse microscopy for regrowth of spindle after cold temperature–induced depolymerization of MTs (30 min in a cold chamber in presence of 1 μM nocodazole) after 72 hours of treatment with control, MLL, WDR5, or Cep72 siRNA. The medium was changed just before imaging after three PBS washes. GFP-tubulin is shown. Time is shown in seconds. Scale bars, 5 μm. See also movies S1 to S4. (**B**) U-2OS cells treated with control, MLL, WDR5, or Cep72 siRNA were observed for MT regrowth after 1 min in mitosis. Cells were stained with α-tubulin (green), PCNT (red), and DAPI (blue). White arrows indicate MT regrowth from the centrosome, and red arrowheads show the MTs originating from the chromosomes. Scale bars, 2 μm. (**C**) Quantification for MT regrowth (from centrosomes) in cells undergoing mitosis is shown upon treatment with control, MLL, WDR5, or Cep72 siRNA. Cells were scored for centrosomes with or without asters, and the percentages of cells are plotted as shown (*n* = 50 centrosomes, *m* = 2 experiments). Error bars represent SEM. *P ≤ 0.01 (two-way ANOVA). (**D** and **E**) Cep72-GFP (green)–expressing cells were acquired in SIM mode after staining with (D) MLL (magenta) and PCNT (red) or (E) MLL (magenta) and WDR5 (red). Zoomed inset (a) shows the multiple intensity projection, and insets (b) and (c) show the 3D full-resolution surface reconstruction of the above SIM images in respective channels or with Cep72-GFP, respectively. The plot shown on the right depicts co-localization profile of MLL with Cep72, which is shown with PCNT (D) or WDR5 (E). Scale bars, 5 μm.

We have previously reported spindle defects upon loss of MLL, which were accompanied with unaligned chromosomes during metaphase (*19*). We questioned whether the underlying cause of spindle defects leading to chromosome misalignment upon loss of MLL/WDR5 is a consequence of impaired MT nucleation during mitosis. To test this hypothesis, we used cells stably expressing GFP–α-tubulin and H2B-mCherry and carried MT regrowth assays in mitotic cells (Fig. 6A and movies S1 to S4). Time-lapse imaging of control cells revealed dense MTs, which appeared almost instantaneously from two centrosomes upon removal of depolymerizing conditions and exhibited spindle formation by 12 s. In contrast, in MLL, WDR5, or Cep72 siRNA-treated cells, nucleation of MTs from centrosomes was attenuated and MT formation continued to be perturbed for the duration of the movie. However, as spindle formation could be seen in MLL/WDR5/Cep72 siRNA-treated cells eventually, we observed for MT nucleation after 1 min after recovery after depolymerization in fixed cells (Fig. 6B). Control cells exhibited dense MTs at both poles, marked by PCNT. In addition, we could observe MTs nucleation from chromosomes. However, upon loss of MLL, WDR5, or Cep72, both spindle poles showed sparse MTs, though MT nucleation from chromosomes continued (Fig. 6B). When quantified for the presence or absence of asters, the number of cells nucleating MTs from centrosome was significantly low in MLL, WDR5, and Cep72 siRNA-treated cells compared to the control (Fig. 6C). In the experiments above, we have checked for spindle regrowth after depolymerization of MTs. To find out whether spindle formation was affected in cells in the absence of any depolymerization agent, we performed time-lapse imaging in siRNA-depleted mitotic cells that were not synchronized using nocodazole treatment. Our time-lapse movies indicate that not only MLL- and WDR5-knockdown cells display sparse MTs but also the spindle poles were not focused (as also reported for Cep72 previously), and, as a result, bipolar spindle could not be formed (fig. S7A and movies S5 to S7) (*32*). Together our results suggest that attenuation of MT nucleation is most affected at the centrosome, but it may be the primary cause of disassembled spindles, resulting in failure of chromosomes to align during mitosis in MLL- and WDR5-depleted cells.

### MLL/WDR5 complex targets Cep72 to the centrosome

Our studies, thus far, suggest that MLL/WDR5/Cep72 act as a complex that is responsible for the recruitment of γ-tubulin, γ-TuRCs, and structural proteins like AKAP9 to the centrosome, and this recruitment ensures proper MT nucleation and regrowth. However, distinct localizations of MLL (Fig. 2) and Cep72 (*32*) warrant further proof that these proteins occur as a complex. First, to orient MLL and Cep72 on the centrosome, we stained Cep72-GFP–expressing cells for MLL and PCNT and performed SIM imaging. As shown in Fig. 6D, PCNT and MLL lie in close proximity to each other. Cep72, although displayed distribution characteristic of a satellite protein, exhibited sufficient overlap with MLL and PCNT on the centrosome. Similarly, when imaged with MLL and WDR5, Cep72 satellites could be seen overlapping with both proteins (Fig. 6E). Notably, MLL and WDR5 displayed remarkable overlap in our SIM analysis (Fig. 6E). Together, our biochemical and SIM data indicate that MLL/WDR5/Cep72 form a complex on the centrosome.

**Fig. 7.**
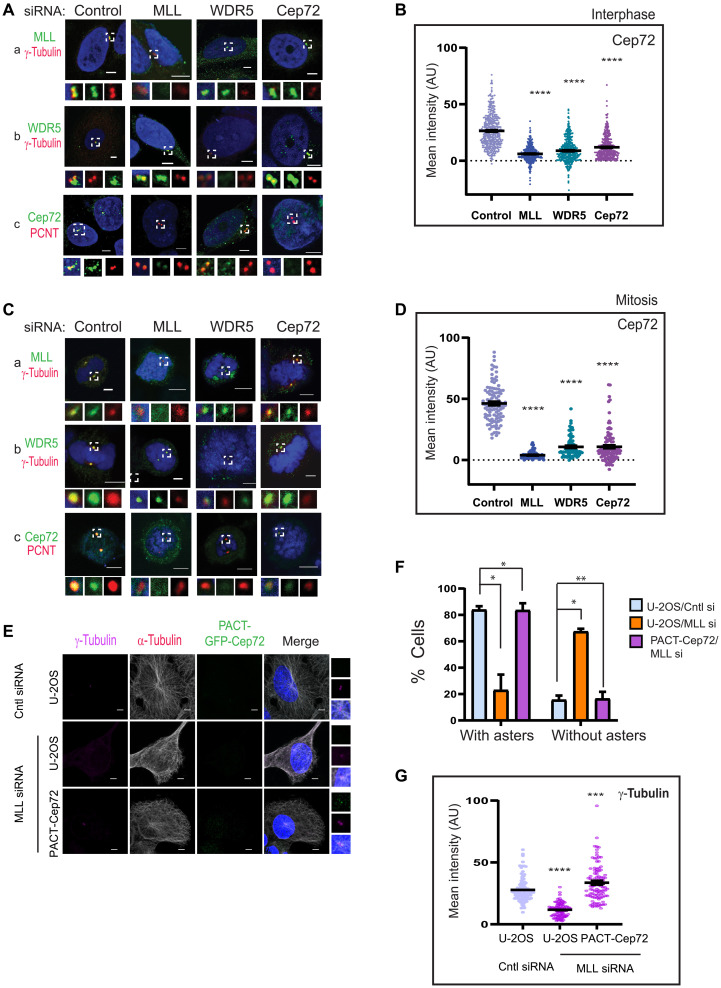
MLL/WDR5 target Cep72 to the centrosome. (**A** and **C**) IFS of U-2OS cells was performed to show Cep72, MLL, and WDR5 levels interphase (A) and mitosis (C) after treating cells with Control, MLL, WDR5, or Cep72 siRNA. Staining for MLL, WDR5, and Cep72 is shown in green, while PCNT and γ-tubulin are shown in red. Scale bars, 5 μm. (**B** and **D**) Quantification for the centrosome intensity of Cep72 is compared for control, MLL, WDR5, or Cep72 siRNA-treated interphase (B) and mitotic (D) cells. Intensity was measured using Zen software, and mean intensities were plotted as shown [(B) *n* = 200 centrosomes, *m* = 2 experiments; and (D) *n* = 50 centrosomes, *m* = 2 experiments]. Error bars represent SEM. ****P ≤ 0.0001 (one-way ANOVA with Bonferroni’s multiple comparisons test). (**E**) Wild-type U-2OS cells or stably expressing PACT-CEP72-GFP were treated with control (Cntl) or MLL siRNA as indicated and stained for γ-tubulin (magenta) and α tubulin (red). Scale bars, 5 μm. (**F** and **G**) Quantification for MT regrowth (F) and γ-tubulin intensity (G) is shown upon treatment with either control or MLL siRNA in U-2OS cells alone or stably expressing PACT-GFP-CEP72 from (E). (F) Cells were scored for centrosomes with or without asters, and the percentages of cells are plotted as shown (*n* = 45, *m* = 2 experiments). (G) γ-Tubulin intensity was measured and plotted from (*n* = 40, *m* = 2 experiments). (E) Error bars represent SEM. **P ≤ 0.0029, ***P ≤ 0.0001, and ****P ≤ 0.0006 (two-way with Šidák’s or one way ANOVA with Tukey’s multiple comparisons test). Scale bars, 5 μm.

Further, we wished to interrogate whether Cep72 recruits MLL/WDR5 complex to the centrosome or vice versa. To answer this question, we treated cells with control, MLL, WDR5, or Cep72 siRNA and determined the effect of this knockdown on centrosome localization of MLL, WDR5, and Cep72 protein. Both in interphase (Fig. 7, A and B) and mitosis (Fig. 7, C and D), the knockdown of Cep72 had no effect on the levels of MLL (Fig. 7, A to D, a) and WDR5 (Fig. 7, A to D, b) at the centrosome, although the levels of γ-tubulin were reduced in the same cells. However, MLL and WDR5 RNAi resulted in a marked loss of Cep72 from the centrosome (Fig. 7, A to D, c) although Cep72 staining could be seen in the cytoplasm (Fig. 7A, c). When quantified, this loss at the centrosome was unexpectedly more than Cep72 siRNA-treated samples (Fig. 7, B and D). Although none of the proteins being analyzed here (Figs. 4 and 5, D and E) show any differential gene expression upon MLL-knockdown or *mll-*null MEFs (*13*, *45*), we further interrogated our findings by overexpressing Cep72-GFP exogenously under a constitutive viral promoter (fig. S7, B and C). Upon MLL or WDR5 siRNA treatment, we observed that Cep72 accumulated around the centrosome like satellite but could not localize to the centrosome like in control cells (fig. S7, B and C). These results mirrored with endogenous Cep72 in MLL_C_D3ΔWIN cell lines, where Cep72 accumulated around the centrosome but could not localize to centrosome, like it did in MLL_C_D3-expressing cells (fig. S7D).

Our above results show that MLL/WDR5 complex recruits Cep72 to the centrosome in addition to the γ-tubulin and γ-TuRCs as shown in Figs. 5 and 7. To show that the loss of γ-TuRCs from the centrosome is a direct consequence of loss of MLL complex anchoring of Cep72, we performed additional experiment where we artificially targeted Cep72 to the centrosome in absence of MLL. We tagged Cep72-GFP with PCNT/AKAP-450 centrosomal–targeting (PACT) domain at the N terminus and stably expressed them in U-2OS cells. After ascertaining that PACT-Cep72-GFP localizes to the centrosome, we treated the cells with MLL siRNA and performed MT regrowth assay (Fig. 7, E and F). The PACT-Cep72-GFP–expressing MLL siRNA-treated cells showed MT regrowth comparable to control cells, indicating that targeting Cep72 to centrosome was sufficient to rescue MT nucleation. This rescue of aster formation at centrosome was accompanied with increased levels of γ-tubulin in PACT-Cep72-GFP–expressing cells (Fig. 7G), indicating that it was the loss of γ-tubulin upon attenuated recruitment of Cep72 that resulted in loss of MT nucleation from the centrosome. Together, our results indicate that MLL and WDR5 are responsible for targeting Cep72 to the centrosome, which, in turn, recruits other MT-nucleating proteins to bring about MT nucleation.

### MT nucleation and Cep72 recruitment are affected in cells derived from patients with WSS

**Fig. 8.**
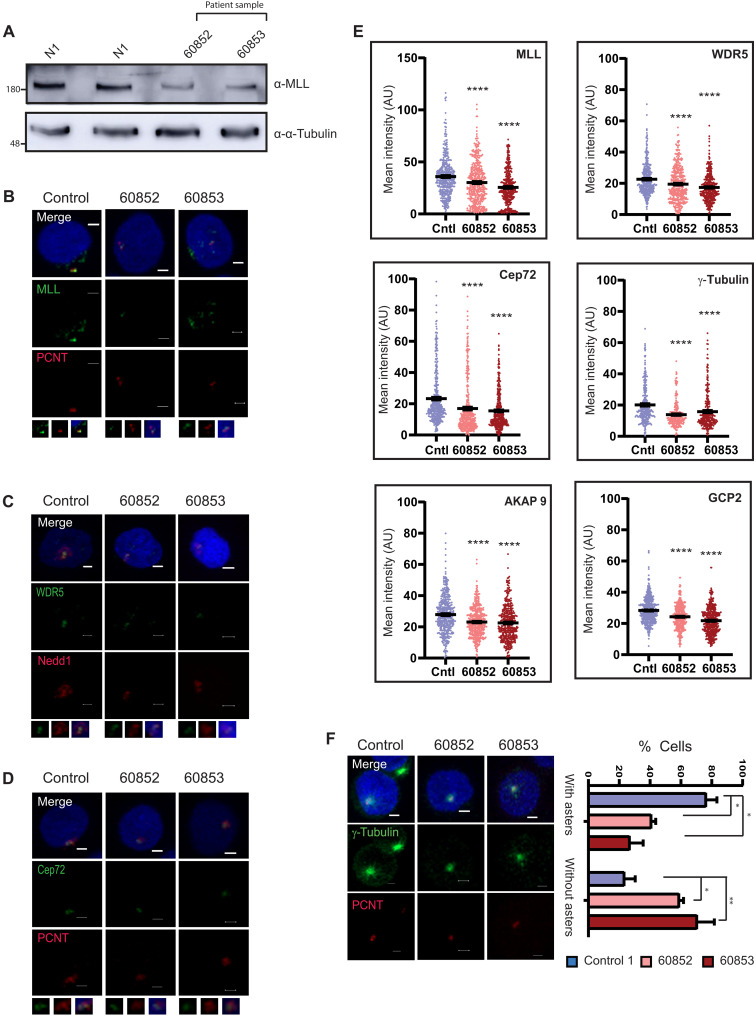
MT nucleation and Cep72 recruitment are affected in cells derived from patients with WSS. (**A**) Western blot analysis of whole-cell lysates shows MLL protein levels in B lymphocytes derived from patients with WSS (60852 and 60853) and two healthy individuals (N1 and N2). Immunoblot was probed with MLL_C_, and tubulin was used as a loading control. (**B** to **D**) IFS of the lymphoblastoid cells of patients (60852 and 60853) and healthy individual (control) using (B) MLL (green), PCNT (red), (C) WDR5 (green), NEDD1(red), (D) Cep72 (green), and PCNT (red) antibodies. Zoomed inset shows the centrosome of the respective cell. Scale bars, 2 μm. (**E**) Centrosome intensities of the indicated proteins were quantified in lymphoblastoid cells derived from patients with WSS and healthy individuals and plotted as shown (*n* = 100, *m* = 4 experiments; for γ-tubulin, *n* ≅ 100, *m* = 3 experiments). Error bars represent SEM. ****P ≤ 0.0001 (one-way ANOVA with Bonferroni’s or Tukey’s multiple comparison). (**F**) MT regrowth assay was performed in lymphoblastoid cell lines (LCLs). MT regrowth was observed through α-tubulin staining (green), and the centrosome was marked with PCNT (red). Quantification of the lymphoblastoid cells showing MT regrowth scored by formation of asters or no asters is shown (*n* ≅ 100, *m* = 2 experiments). Error bars represent SEM. *P ≤ 0.03 and **P ≤ 0.0069 (two-way ANOVA with uncorrected Fisher’s least significant difference). Scale bars, 2 μm.

We obtained B lymphocytes from two patients with WSS who carry the premature stop codon 3247C>T (p. Arg1083*) in exon 4 of *MLL*/*KMT2A* gene, resulting in nonsense-mediated mRNA decay (*47*). Both the patients were monozygotic twins carrying the same mutation as confirmed by Sanger sequencing (referred to as 60852 and 60853) (*47*). Western blots with MLL antibody against the C terminus revealed ~50% reduction in the level of MLL protein when compared with samples from healthy individuals (N1 and N2; Fig. 8A). We could observe MLL, WDR5, and Cep72 localizing to centrosome with other canonical centrosome proteins (Fig. 8, B to D). Consistent with the reduced total protein levels of MLL, the intensity of MLL on the centrosome also showed a decrease in patient cells (Fig. 8, B and E). Unexpectedly, WDR5 levels also showed a slight but consistent decrease in both 60852 and 60853 samples (Fig. 8, C and E). In line with our hypothesis that MLL recruits Cep72 to the centrosome, the protein levels of Cep72, γ-tubulin, NEDD1, GCP2, and AKAP9 were decreased in the patient lymphoblastoid cells (Fig. 8, B to E, and fig. S8, A to C). We observed a significant decrease in PCNT also (Fig. 8, B, D, and E, and fig. S8C). To test whether other structural proteins are affected by reduced MLL levels, we stained and quantified CDK5RAP2, another microcephaly-associated structural component of PCM (*48*, *49*). Unlike PCNT and AKAP9, CDK5RAP2 levels were unchanged on the centrosome (fig. S8, B and C). All in all, consistent with our experiments in cancer cells, lymphoblastoid cells derived from patients with WSS also showed diminished recruitment of Cep72 and its associated proteins involved in MT nucleation.

To test whether this reduced level of centrosome proteins has a functional consequence, we performed regrowth assays on the lymphoblastoid cells and observed that MT nucleation and aster formation were dampened (Fig. 8F). We checked for protein levels of the key proteins examined here and found that, except MLL, all other protein levels are largely unchanged (fig. S8D). Together, our results imply that MLL has a crucial developmental role in the functions of centrosomes.

## DISCUSSION

Our work assigns a critical functional role to the MLL/WDR5 complex at the centrosome in promoting the MT nucleation in both interphase and mitosis. The present study not only outlines the combinational role of MLL/WDR5 in centrosome MT regrowth but also delineates how the centriolar satellite protein, Cep72, a key protein required in MT nucleation activity, is recruited to the centrosome.

### MLL is a part of the centrosomes

Centrosomes are non-membranous organelles that are well accepted to be the major MT-organizing center and a signaling hub in a cell, channeling protein transports, maintaining cell shape, cell polarity in addition to spindle apparatus formation, and proper chromosome segregation (*50*, *51*). On the flip side, they form primary cilium in G1/G0 arrested, or differentiated cells and help in cell migration and intercellular cross-talk (*52*, *53*). Here, consistent with our previous observations, we report that both MLL and WDR5 localize to the centrosomes throughout the cell cycle (*19*, *20*). In line with our observations here, we have previously reported that knockdown of MLL or WDR5 increases the prevalence of mitotic defects like impaired chromosome segregation, likely leading to aneuploidy, a hallmark of centrosome dysfunction (*19*, *38*).

Structural and other proteins, arranged in a hierarchically organized order in the PCM, give the centrosome its structural and functional stability. We observed that MLL/WDR5 co-localized with the PCM protein, γ-tubulin, in all the phases of the cell cycle including mitosis, stained persistently on the centrosomes even upon depolymerization of MTs by the cold treatment, and dissociated from the centrosome upon Taxol treatment, indicating that both these proteins are bona fide components of the centrosomes. Notably, MLL/WDR5 staining was different from the localization pattern of the centriolar satellite protein, Cep72. Our SIM analysis further showed that MLL localized in the proximity of centrioles, within the PCM (marked with PCNT and γ-tubulin staining). All these observations reinforce that MLL/WDR5 complex is associated with the protein pool of centrosomal PCM.

Most of the H3K4 HMT literature focuses on the roles of these complexes in the nucleus, and less is known regarding the cytoplasmic localization and function of H3K4 HMT subunits (*54*, *55*). MLL being a histone modifier harbors different domains related to its chromatin binding and methylation activity. Here, we show that the MT nucleation defects arising in the absence of MLL are independent of its methylation activity. Previously, other histone modifiers have been reported to localize on the centrosome and perform various centrosome-related functions. For instance, histone acetyltransferases KAT2A and KAT2B and histone deacetylase (HDAC) SIRT1 are involved in centrosome duplication, while HDAC8 is involved in centrosome cohesion. These proteins affect the centrosomal functions not by altering gene expression but by directly interacting with centrosome kinases like polo-like kinase (*56*–*58*). Cytoplasmic roles of MLL complex proteins are also reported previously (*19*, *20*, *59*). Earlier studies also suggest that the SET-domain functions of MLL do not play a crucial role in the development or the control of cell division (*11*, *38*, *60*).

In line with these observations, complementation experiments indicated that the MLL activity at the centrosome was found to be independent of the SET domain but dependent on its WDR5 interaction, as the WIN motif–mutant construct of MLL was able to localize to centrosome but was incapable of nucleating MTs from the centrosome. WDR5, by virtue of its WD40 β-propellers, can perform a scaffolding role (*61*). In support of this hypothesis, WDR5 F133L mutant is incapable of MT nucleation. Even MLL, with its large structure and coiled coil domains, has the potential to act as a scaffold to bring various proteins to the PCM. Although we could not detect a clear WIN motif, Cep72 was listed as one of the proteins harboring atypical N-terminal WIN motif in a recent study (*62*). Together, our current observations shed light on one of the potential SET domain–independent roles of MLL in the functioning of the centrosomes and the diseases caused by centrosomal dysfunction.

### MLL regulates centrosome MT nucleation

Here, we show that defects in MT nucleation and regrowth upon loss of MLL/WDR5 are the causal effects of mis-localization of Cep72 protein. Cep72 controls nucleation by affecting the localization of γ-tubulin, GCP2, and AKAP9, proteins whose roles in MT nucleation are already reported (*26*, *63*). In line with the above reports and consistent with our hypothesis that the MLL/WDR5 complex administers the recruitment of Cep72, the localization of γ-tubulin, GCP2, AKAP9, and Cep72 was affected on the centrosome upon MLL/WDR5 depletion. Thus, MLL/WDR5 function upstream of Cep72 and affect the seeding template required for the rapid MT nucleation. This phenotype was prominent in mitosis as centrosome-specific MT nucleation was affected causing unstable and unfocused spindle poles (*32*) (this study). In line with these observations, depletion of both Cep72 and MLL/WDR5 has been reported to cause multipolar spindles and defective chromosome alignment by independent studies (*19*, *32*) (this study). As previously reported for Cep72 depletion, we also observed that more than two PCNT fragments were present in about 40% of MLL or WDR5 siRNA-treated cells. However, it has been proposed that mitotic delay, including those induced by nocodazole, causes a time-dependent increase in centrosome fragmentation (*64*). As we used nocodazole for synchronization in our experiments and have reported considerable mitotic delay upon MLL and WDR5 RNAi (*19*), we are not sure whether the appearance of PCM fragmentation is a direct or indirect effect of MLL/WDR5 depletion. Still, despite observing PCM fragmentation, we did not observe diffuse γ-tubulin as reported before (*32*). These differences in centrosome localization patterns have also been reported for other protein such as CDK5RAP2, which shows fibrous staining radiating from the centrosomes in MEFs, which was not seen in NIH-3T3 (*65*). Therefore, differences in antibodies, cells used, and imaging platforms may explain the different observations in both studies (*32*) (this study). In particular, the duration of our RNAi (60 hours versus 72 hours) may have a greater impact on the appearance and intensity of various phenotypes observed here.

Cep72 has been shown to affect the localization of structural protein AKAP9 and CDK5RAP2 but not PCNT (*23*, *29*, *32*, *33*, *66*). Of these three structural proteins, the role of AKAP9 in centrosome-mediated nucleation is underappreciated. Although AKAP9 depletion does not affect γ-tubulin levels at the PCM, it is known to recruit γ-TuRCs (GCP2/3) and can initiate MT nucleation at the centrosome through a pathway distinct from its Golgi-based MT nucleation pathway (*29*, *67*). Although we did not check at the Golgi, we observed a marked reduction in the levels of AKAP9 at the centrosome upon depletion of MLL/WDR5 both in interphase and mitosis with a concatenate decrease in MT nucleation. In contrast, PCNT was differently affected in different stages/cells in our experiments. While PCNT levels in interphase U-2OS cells and MEFs remained unchanged, they were notably affected in mitosis. Distinct from these observations, the patient lymphoblastoid cells showed a small but significant reduction in PCNT levels. PCNT and γ-tubulin are reported in several different pools at the centrosome. Distinct from the universal γ-TURCs, PCNT and γ-tubulin formed a complex and played a role in centrosome MT nucleation in the Xenopus egg extracts (*68*). Moreover, this complex is mitosis specific (*68*, *69*). Another report showed that PCNT is recruited to the centrosome by PCM-1 in a dynein-dependent manner, but not γ-tubulin (*70*). To reconcile these different observations, a hypothesis has been put that γ-tubulin may exist in different pools at the centrosome (*71*, *72*). MLL/WDR5 complex has been shown to interact with dynein, but the functional implications of this interaction are not known (*19*). All the observations listed above may obliquely explain the differential effect of MLL/WDR5 depletion on the recruitment of PCNT in our experiments. Among the list of proteins co-purified with γ-TURCs, NEDD1 depletion also affects recruitment of γ-TURCs, making the centrosomes incapable of MT nucleation (*44*). Our results indicate that MLL/WDR5 function upstream of NEDD1, affecting its localization to centrosomes both in interphase and mitosis, potentially regulating another branch of MT nucleation pathway. The recruitment of γ-tubulin by NEDD1 is shown to be mediated by the direct interaction of these two proteins and also by the recruitment of NEDD1 to the centrosomes by other centrosomal maturation proteins, e.g., Cep192, further indicating the possibility of multiple regulatory pathways for centrosome MT nucleation (*73*). Last but not least, all three structural proteins, i.e., PCNT, AKAP9, and CDK5RAP2, interact with each other in some form or another, more often in mitosis (*29*, *74*, *75*), and hence may affect each other’s accumulation on PCM indirectly. Our findings along with the previous reports may suggest the involvement of MLL/WDR5 in more than one MT-nucleating pathways or they may regulate a certain pool of centrosome-nucleating proteins during interphase, and, as the cell progresses toward mitosis and the centrosome starts maturating to play the central role of nucleation and spindle formation, the role of MLL/WDR5 may also extend to regulate other pools of structural proteins to ensure proper centrosome nucleation, spindle formation, and, ultimately, correct cell division. It will be interesting to find the common links, if any, between these multiple MT nucleation regulatory pathways at the centrosome.

### Recruiting a centriolar satellite protein

Centrosome proteins are known to require centriolar satellite proteins like pericentriolar material 1 (PCM-1), CEP72, and CEP90 for their optimal enrichment at the centrosomes, but, in return, whether pericentriolar proteins play any role in the recruitment of centriolar satellite proteins has not been reported (*33*, *46*, *70*). Here, we have shown that proteins that are part of PCM like MLL/WDR5 also have functional relevance for the localization of centriolar satellites like Cep72. It is remarkable to note that, despite substantial efforts to understand the composition and interactions of satellite-associated proteins, the exact mechanism behind the assembly and localization of centriolar satellites remains unclear. Two separate hypotheses implicating MT and/or PCM-1 have been put forward for the assembly and localization of centriolar satellites to the centrosome. A handful of studies conducted on PCM-1 have led to the proposal that, besides facilitating the transport of satellites toward the centrosomes in a minus-end-directed manner, MTs also have a role in the assembly of satellites (*70*, *76*–*78*). However, whether the MT-based transport is relevant for other satellite proteins remains to be tested. Furthermore, recent studies have shown that the composition of satellites remains mostly unaffected by the disruption of MTs (*79*).

Similarly, PCM-1 is proposed to be important for the assembly and localization of centriolar satellites (*70*, *80*). However, upon loss of PCM-1, the centrosomal levels of most satellite proteins were unchanged (*81*). Furthermore, a substantial number of interactions between satellite proteins continue to exist in the absence of PCM-1 (*79*). The satellite proteins, whose localization was dependent on PCM-1, lost satellite formation but not centrosome targeting (*79*). These findings indicate that complexes of satellite proteins can assemble independently of PCM-1. We propose that PCM-based proteins like MLL/WDR5 are involved in the recruitment of centriolar satellites, particularly in targeting them from the satellite to the centrosome. In our experiments, upon MLL and WDR5 depletion, centrosomal targeting of endogenous Cep72 was severely affected. Cep72 overexpression led to concentrated staining of Cep72 around the centrosomes, but it could not localize to the centrosome in the absence of MLL or WDR5. We propose that multiple actors like PCM-1 and MLL/WDR5 are involved in targeting distinct complexes of satellite proteins to the centrosome. In support of our hypothesis, MLL and/or WDR5 occur as interacting partners of several centriolar satellite proteins (*79*), underscoring their yet undiscovered role in the assembly of satellite proteins.

### Role of MLL in WSS

Centrosomal defects in neuroblasts affect their division and normal brain development (*82*). Genetic mutations in centrosome-associated proteins like CDK5RAP2 and abnormal spindle-like, microcephaly-associated (ASPM) (*48*, *83*) lead to microcephaly. Recently, it has been shown that Cep72 along with other satellite proteins is involved in targeting microcephaly proteins (*33*). Although the pathogenicity of the *MLL* gene as a cause of WSS has been known for some time, how exactly it contributes to the development of the disease is not known (*84*). A large percentage of mutations reported are nonsense or large deletions where mutant transcript undergoes mRNA decay, affirming that haploinsufficiency of MLL is the main pathogenic mechanism. The patients with WSS present a variety of phenotypes including hypertrichosis cubiti, characteristic facial features, psychomotor delay, and microcephaly. Mild to moderate growth retardation, developmental delay, and intellectual disability are commonly observed in this syndrome, while microcephaly has been reported in 30 to 60% of the patients. Consistent with these phenotypes, studies in mice have reported a role of MLL in synaptic plasticity, cognition, and long-term memory attributing it to loss of H3K4 methylation at modest 50 loci in the neurons (*85*). We propose that functions of MLL at the centrosome are likely to bear a greater impact in the development of brain. Here, we link the functional role of MLL/WDR5 at the centrosomes to the WSS. Consistent with our hypothesis, B lymphocytes from patients with WSS showed reduced localization of all the major structural and functional centrosomal proteins such as AKAP9, NEDD1, γ-tubulin, GCP2, and Cep72, as demonstrated in the cancer cell line upon depletion of MLL; thus, our results are biomimetic. Consistent with the defects in the recruitment of key proteins, MT nucleation was also slower in the patient B lymphocytes, raising the possibility that multiple key processes may be affected (*86*). While it is probable that multiple roles of MLL are required during the development of an organism, our data present possibilities that activities other than transcription/methylation, like those described here, may contribute to the functions of MLL in development and disease.

## MATERIALS AND METHODS

### Cell culture

U-2OS (human osteosarcoma), HeLa (human cervical adenocarcinoma) and IMR-90 tert (telomerase-immortalized IMR-90 human) cells, HeLa-Flp-In cells (a gift from S. S. Taylor), and MEFs (a gift from Y. Dou) were grown in Dulbecco’s modified Eagle’s medium supplemented with 10% fetal bovine serum, l-glutamine, and penicillin/streptomycin at 37°C. All cell lines were authenticated by Lifecode Technologies Private Limited (India). Lymphoblastoid cell lines (LCLs) were maintained in RPMI medium with 10% fetal bovine serum, l-glutamine, and penicillin/streptomycin at 37°C in T25 or T75 vented flasks. To induce MLL deletion in MEFs (*Mll1*^f/f^; *ER-Cre*^+/−^), the cells were treated with 4-hydroxytamoxifen (Sigma-Aldrich, H7904) to a final concentration of 100 nM for 48 hours, and the protein levels were confirmed by the SDS–polyacrylamide gel electrophoresis (PAGE) and Western blot analysis.

### Cloning and site-directed mutagenesis

cDNA constructs expressing full-length MLL and its deletions, −WDR5, and GST-WDR5 F331A have been described before (*19*, *38*). pGEX4t1 was linearized with Xho I, and polymerase chain reaction (PCR)–amplified MLL fragments were cloned to generate GST-MLL_C_D1 (2723 to 3092 amino acids), GST-MLL_C_D2a (3084 to 3445 amino acids), GST-MLL_C_D2b (3448 to 3694 amino acids), and GST-MLL_C_D3 (3694 to 3969 amino acids). MLL fragments were PCR amplified and cloned into GFP-tagged Xho I–linearized pcDNA3 (puro) vector, giving rise to GFP-MLL_C_D1 (2718 to 3275 amino acids), GFP-MLL_C_D2 (3281 to 3740 amino acids), and GFP-MLL_C_D3 (3741 to 3969 amino acids). MLL_C_D3 (3741 to 3969 amino acids) was cloned into S protein, FLAG, and streptavidin-binding peptide (SFB)–tagged pcDNA5 vector after Xho I linearization. All the PCR cloning was performed using the In-Fusion HD Cloning Kit (Takara) as per the manufacturer’s instructions. pcDNA-SFB-MLL_C_D3Δwin (R3765A) point mutation was obtained by site-directed mutagenesis PCR. Full-length Cep72 was generated by reverse transcription PCR using RNA prepared from U-2OS cells and cloned into GFP-tagged pcDNA3 (puro) and pGEX4t1 vectors. All PCR-based clones and mutations generated were verified by sequencing the entire cDNA inserts. pcDNA-PACT-Cep72-GFP was generated by PCR amplifying the PACT domain and cloning it to the N terminus of Cep72-GFP (the PACT domain of PCNT was gifted by M. Schuh).

### Stable cell line generation

Generation of cell lines expressing FLAG-tagged MLL and MLL deletions have been described before (*38*). U-2OS cell lines expressing GFP-MLLc fragments D1 to D3, Cep72-GFP, and GFP–Centrin-2 (Addgene, plasmid 41147) were generated by transfecting corresponding plasmids using Lipofectamine 2000 (Invitrogen), selected in puromycin (4 μg/ml; Thermo Fisher Scientific), and maintained in puromycin (2 μg/ml). Enhanced GFP (EGFP)–tubulin and H2B-mCherry cell lines were obtained by transfecting EGFP-tubulin (Addgene, plasmid 30487) in U-2OS cells already expressing H2B-mCherry (gift from M. Overholtzer) and were selected in G418 (Thermo Fisher Scientific) at concentration (200 μg/ml). HeLa Flp-In cells expressing pcDNA-SFB-MLL_C_D3 and pcDNA-SFB-MLL_C_D3Δwin (R3765A) were generated as described before (*87*).

### RNA interference

The sequences of MLL1, WDR5, Cep72, Kif2A, and Luciferase (control) siRNAs used have been described elsewhere (*19*, *32*, *38*). siRNA transfections were performed using Oligofectamine as described before (*88*). Treated cells were harvested 72 or 96 hours after transfection and were used for subsequent experiments.

### Generation of LCLs

Five milliliters of blood was collected from patients and healthy individuals after informed signed consent was obtained from parents. The study was conducted in accordance with the provisions of the Declaration of Helsinki. All experiments were performed after being approved by the Centre for DNA Fingerprinting and Diagnostics (CDFD) institutional ethics committee (approval number IEC 39 and 40).

To immortalize an individual’s B lymphocytes in continuous culture for experimental use, the peripheral blood mononuclear cells of healthy individuals or patients were isolated from the blood samples using the density gradient centrifugation over Histopaque (Sigma-Aldrich, 10771) and infected with Epstein-Barr virus obtained from the marmoset LCL B95.8 grown in RPMI medium in 1:1 ratio for 24 hours in an upright T25 flask. Cyclosporin A (200 ng/ml; Sigma-Aldrich, C3662) was added during the viral infection to suppress the B-cell receptor-mediated lytic induction of the virus. After 24 hours, the viral medium was replaced with the normal RPMI medium, and cells were allowed to grow undisturbed for 3 to 4 weeks until they gave rise to continuously proliferating LCLs, which were used for further experimentation.

### Protein expression

All the GST-tagged proteins except GST-Cep72 were expressed in BL21(DE3) *Escherichia coli* strain in LB broth. GST-Cep72 was expressed in Rosetta Gami (DE3) *E. coli* strain in Terrific Broth. The cells were induced by adding 0.2 mM isopropyl β-d-thiogalactopyranoside at 18°C. After induction, they were lysed in lysis buffer [50 mM tris (pH 7.4), 150 mM NaCl, 0.1% NP-40, 1 mM dithiothreitol (DTT), and 1 mM phenylmethylsulfonyl fluoride (PMSF)], incubated with glutathione agarose beads (Sigma-Aldrich) at 4°C for 3 hours. The beads were harvested and washed three times with wash buffer [50 mM tris (pH 7.4), 500 mM NaCl, 0.1% NP-40, 1 mM DTT, and 1 mM PMSF]. Protein concentration was estimated by SDS-PAGE followed by Coomassie brilliant blue staining.

### Immunoprecipitation and GST affinity pull-downs

Immunoprecipitation of endogenous proteins was performed using anti-MLL_C_ (05-765, EMD Millipore) or anti-WDR5 (A302-430A, Bethyl Labs) or anti-Cep72 (A301-297A, Bethyl Labs) antibody overnight at 4°C incubated with precleared HeLa cell lysate prepared in lysis buffer [100 mM NaCl, 20 mM tris (pH 7.4), 0.5 mM EDTA, 0.5% NP-40, 0.5 mM PMSF, 1 mM DTT, aprotenin (1 μg/ml), and pepstatin (1 μg/ml)], followed by incubation with Protein A Sepharose beads (Cytiva) for 2 hours. The beads were washed three times in wash buffer [15 mM tris (pH 7.4), 100 mM KCl, 0.02% NP-40, 1 mM DTT, and 1 mM PMSF], and bound proteins were then subjected to SDS-PAGE and detected by Western blotting. For GST affinity pull-downs, bead-bound purified proteins were incubated with HeLa or U-2OS whole-cell lysate (prepared as described above) for 3 hours and washed three times in wash buffer (described above), and bound proteins were subjected to SDS-PAGE followed by the Western blotting.

### Western blot

The samples were lysed in nuclear and cytoplasmic extraction (NETN) buffer [20 mM tris (pH 8.0), 100 mM NaCl, 0.5 mM EDTA, 1% NP-40, and protease inhibitor cocktail], and protein concentration was estimated using Bradford’s method. Protein (100 μg) from each sample was boiled and electrophoresed on SDS-PAGE gel, transferred on polyvinylidene difluoride membrane (Amersham), and incubated with primary antibodies (anti-MLL1: A300-374A and A300-086A, Bethyl Labs; anti-WDR5: A310-880A, Bethyl Labs; anti-PCNT: ab4448, Abcam; anti-NEDD1: ab57336, Abcam; anti–γ-tubulin: GTU-88, T6557, Sigma-Aldrich; anti–α-tubulin: T9026, Sigma-Aldrich; anti-Cep72: A301-297A, Bethyl Labs; anti-Cep72: 19928-1-AP, Proteintech; anti-GCP2: PA5-21433, Thermo Fisher Scientific; anti-AKAP9: 611518, BD Laboratories; and anti-CDK5RAP2: ab70213, Abcam). The immunoblots were scanned on Odyssey infrared imager (LI-COR) using secondary antibodies (goat anti-mouse IRDye 800CW, 926-32210; goat anti-mouse IRDye 680LT, 926-68020; and goat anti-rabbit IRDye 680LT, 926-68021) or ImageQuant LAS500 (GE Healthcare) system using horseradish peroxidase–conjugated anti-mouse (Bio-Rad, 170-6516) or rabbit (Bio-Rad, 170-6515) secondary antibodies. See fig. S9 for whole blots used in the figures.

### MT regrowth assay

To perform this assay, the siRNA-transfected cells were kept in ice-cold medium for 1 hour after 72 hours of siRNA transfections. After 1 hour of cold treatment, the medium was replaced with 37°C pre-warmed medium for 0/1/5/15/25 min to allow MT nucleation and regrowth. These cells were fixed at the indicated time point in −20°C chilled methanol for 1 min and scored for aster formation by microscopy. To obtain the mitotic cells, U-2OS cells were treated additionally with nocodazole (100 ng/ml) for 12 hours after 60 hours of siRNA treatment. For Taxol experiment, U-2OS cells were incubated with 5 μM paclitaxel (Sigma-Aldrich, T7402) for 24 hours, washed thrice with phosphate-buffered saline (PBS) and fixed with acetone for 90 s. For time-lapse imaging, to depolymerize MTs by cold treatment, U-2OS cells were incubated in ice cold chamber for 30 min with medium containing 1 μM nocodazole, washed thrice with PBS, incubated with warm medium, and imaged live.

To perform MT regrowth assay in LCLs, the cells are seeded on fibronectin (50 μg/ml)–treated coverslips and incubated at 37°C for 1 hour, allowing cells to attach. After 1 hour, the culture was transferred to cold treatment for 1 hour followed by incubation with 37°C pre-warmed medium for 1 min. The cells were fixed in −20°C chilled methanol for 1 min and scored for aster formation by microscopy.

### Immunofluorescence microscopy

U-2OS cells or MEFs were grown on glass coverslips and fixed with acetone chilled at −20°C for 60 or 90 s (at −20°C) for centrosome localization. Fixation for MT regrowth assay has been described above. The lymphoblastoid cells were first spun down at 100*g* for 5 min. The cells were then seeded on the coverslips for 10 min in 1× PBS at room temperature and fixed with 1% paraformaldehyde for another 10 min. After three washes of 5 min each with 1× PBS, permeabilization was done with 0.5% triton-X in 1× PBS for 10 min. In case of CDK5RAP2 and γ-tubulin staining in the LCLs, the cells were fixed with chilled methanol on ice for 1 min. Cells were blocked using 1% bovine serum albumin for 1 hour at room temperature and stained with primary antibodies [anti-MLL1: A300-374A and A300-086A, Bethyl Labs; anti-MLL_C_D1 (*89*); anti-WDR5: A310-880A, Bethyl Labs; anti-PCNT: ab4448 and ab28144, Abcam; anti-NEDD1: ab57336, Abcam; anti–γ-tubulin: GTU-88, T6557, Sigma-Aldrich; anti–α-tubulin: T9026, Sigma-Aldrich; anti-Cep72: A301-297A, Bethyl Labs; anti-Cep72: 19928-1-AP, Proteintech; anti-GCP2: PA5-21433, Thermo Fisher Scientific; anti-AKAP9: 611518, BD Laboratories; anti-CDK5RAP2: ab70213; and anti-FLAG: F7425, Sigma-Aldrich] and conjugated secondary antibodies (Alexa Fluor 488: A11029 and A11034; Alexa Fluor 568: A11031 and A11036; Alexa Fluor 594: A11032 and A11037; or Alexa Fluor 633, A21070 and A21050, Invitrogen). Immunofluorescence images were captured on ZIESS LSM 700 or 900 inverted confocal microscope using 63× objective lens. Data analysis was carried out using ZEN BLUE2/ZEN BLACK software. For quantifications, the mean intensities were calculated using ZEN software. A defined circular graphical element (region of interest) was drawn around the centrosome to measure centrosome intensity and near the centrosome to measure the background intensity. The centrosomal mean intensity was then obtained by subtracting the respective background intensity to get the plotted absolute mean intensity value. The numbers of data points acquired and experiments performed are mentioned in the legends.

For SIM images, cells were grown on coverslips, fixed with −20 chilled methanol for 90 s or 2 min and were processed as described for confocal microscopy. Images were captured on ZEISS Elyra 7 with Lattice SIM^2^ microscope using 63× objective lens. 3D-SIM images were reconstructed by Lattice SIM 3D processing using ZEN 3.0 SR software. Time-lapse imaging was performed using U-2OS cells stably expressing GFP-tubulin and H2B-mCherry. Cells were cultured in 35-mm glass bottom dishes (Arrow Labs) and were imaged at ×40 magnification on NIKON ECLIPSE Ti-E inverted microscope or on Zeiss LSM 700 or 900 META inverted confocal microscope by acquiring one frame per minute. The time-lapse movies in fig. S4D were subjected to deconvolution using AutoQuantX3 and analyzed using NIS-Elements and Fiji software.

### Statistical analysis

For statistical analysis, GraphPad Prism software was used. Tukey’s test, Bonferroni’s test, unpaired Student’s *t* test, and one-way and two-way analysis of variance (ANOVA) were used for different experiments as stated in the figure legends. Error bars represent SEM. See table S1 for raw data points used in the figures.
