# Supplementary material for: MLL/WDR5 complex recruits centriolar satellite protein Cep72 to regulate microtubule nucleation and spindle formation
Source: Sci Adv. 2024 Dec 11;10(50):eadn0086. doi: 10.1126/sciadv.adn0086 (PMC11633745; doi:10.1126/sciadv.adn0086)
Supplement: Supplementary file 1 — Figs. S1 to S9 Legend for table S1 Legends for movies S1 to S7 [file sciadv.adn0086_sm.pdf]

Science Advances
AAAS

Supplementary Materials for

MLL/WDR5 complex recruits centriolar satellite protein Cep72 to regulate microtubule nucleation and spindle formation

Swathi Chodisetty *et al.*

Corresponding author: Shweta Tyagi, shweta@cdfd.org.in

Sci. Adv. **10**, eadn0086 (2024)
DOI: 10.1126/sciadv.adn0086

The PDF file includes:

Figs. S1 to S9
Legend for table S1
Legends for movies S1 to S7

Other Supplementary Material for this manuscript includes the following:

Table S1
Movies S1 to S7

Supplementary Figure Legends:

Figure S1: The MLL complex localizes to the centrosomes.

A) U-2OS cells were labelled with α-MLL$_N$ (green) and α-γ-tubulin (red) to visualize the centrosomal localization of endogenous MLL during the specific cell cycle stages. DNA stained with DAPI is shown in blue. Scale bar=5μm.

B-C) U-2OS cells were treated with two different siRNAs for MLL (#1 and #2) or WDR5 (#1 and #2) and, Western blot analysis of whole cell lysate of each sample is shown. Immunoblots were probed with **B)** α-MLL and **C)** α-WDR5; α-tubulin was used as a loading control. The numbers on the left indicate the position of molecular weight markers (in kDa).

D-E) IFS was performed in U-2OS cells, 72 hr post Control, MLL siRNA (**D**) or WDR5 siRNA treatment (**E**) and stained with PCNT (red) and MLL(**D**) and WDR5(**E**) (green). Error bars represent the SEM . ****$P \leq 0.0001$, **$P \leq 0.0025$, ns: not significant (ordinary one-way ANNOVA with Sidak's or Tukey's multiple comparison). (MLL: $n \cong 80$ centrosomes, $m=2$ experiments; WDR5: $n \cong 150$ centrosomes, $m=2$ experiments). Scale bar=5μm

F) IFS was performed in wild-type ($mll1^{+/+}$) and *mll* null ($mll^{-/-}$) mouse embryonic fibroblasts (MEFs) after 48hr of OHT treatment, and cells were stained for PCNT (red) and MLL (green). Scale bar=5μm. The mean intensities of the centrosomal staining were plotted as shown. Error bars represent the SEM. , ****$P \leq 0.0001$, **$P \leq 0.0025$ ns: not significant (ordinary one-way ANNOVA with Sidak's or Tukey's multiple comparison). ($n \cong 100$ centrosomes, $m=2$ experiments)

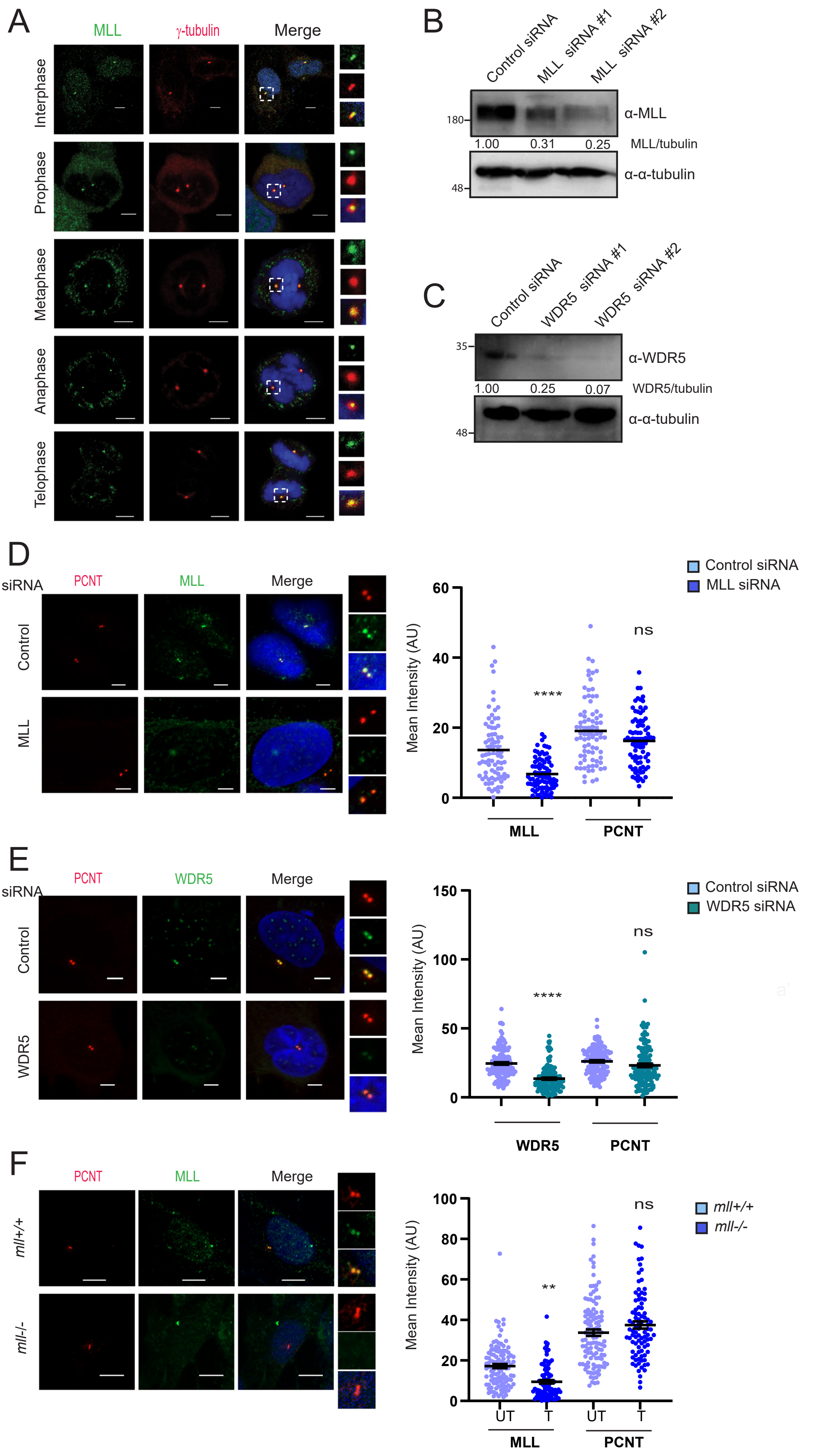

Supplementary Figure 1

Figure S2: The MLL complex is integral to the centrosomes.

A) U-2OS cell are stained with α-α-tubulin (a, b), α-MLL (c, d) or WDR5 (e, f) before and after the cold treatment on ice for one hour to visualize the effect of MT depolymerization on the localization of MLL and WDR5 (shown in green) on the centrosomes. γ-tubulin is used to mark the centrosomes (red). The zoomed inset corresponds to the white box in the respective panels. Scale bar=5μm.

B) MLL, WDR5 and γ-tubulin levels were quantified before and after cold treatment as shown in **A (***n*=150, *m*=2 experiments**)**. Error bars represent SEM. ****$P\leq 0.0001$, ns: not significant (two-way ANOVA).

C) Distribution of WDR5 (purple) on the centrosomes was compared in taxol-treated, and -untreated U-2OS cells. Pericentrin (PCNT, gray) and GFP-Centrin were used as positive control. (**A-C**) Scale bar= 5μm. Zoomed-in images are shown in the inset.

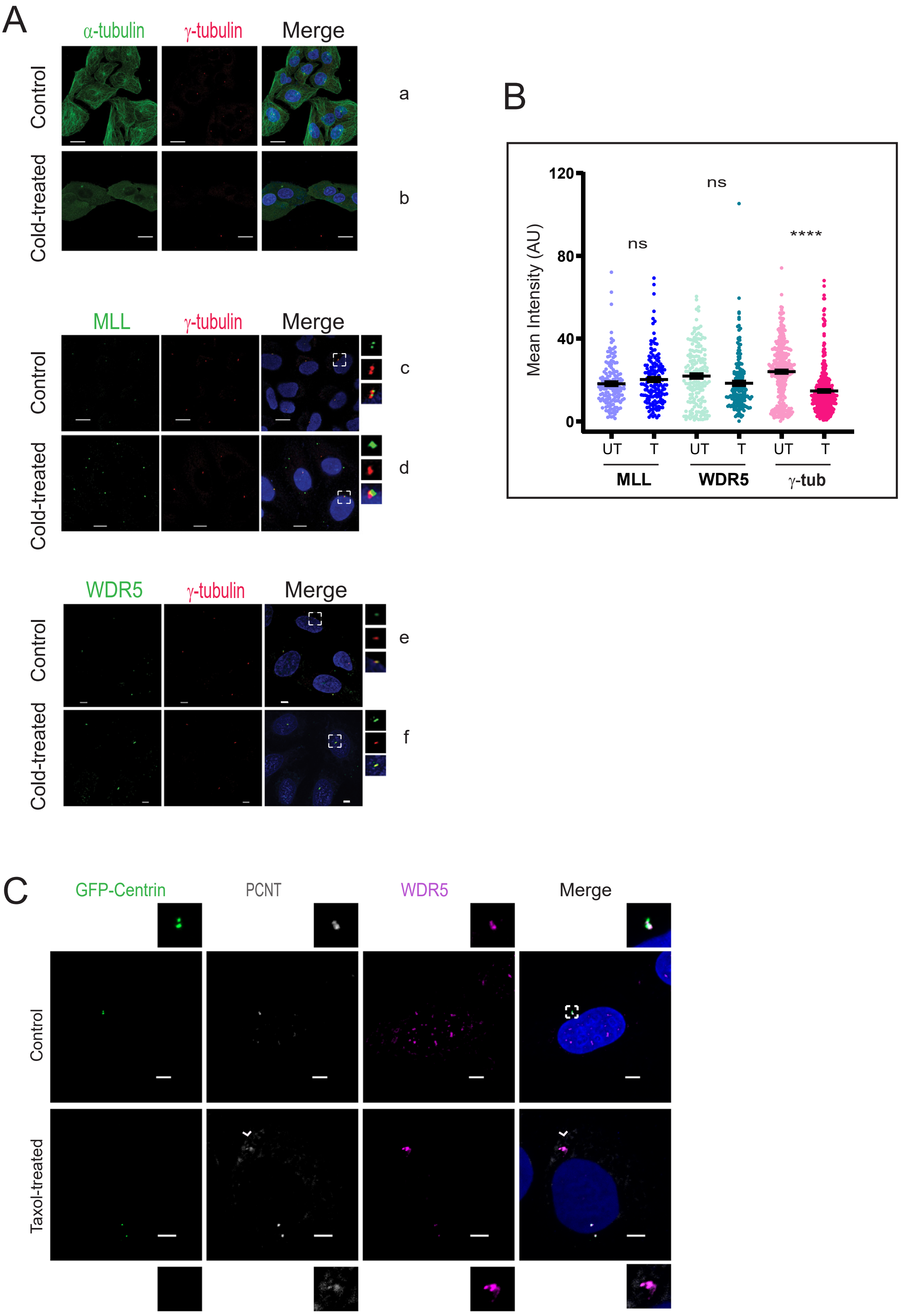

Supplementary Figure 2

Figure S3: MLL and WDR5 regulate microtubule nucleation and regrowth

A) U-2OS cells stably expressing GFP-Centrin were stained with different dilutions of α-MLL(1:50, 1:100 and 1:200) to visualize the MLL localization at the centrosomes. Zoomed inset (a) shows the multiple intensity projection and inset (b, c) shows the 3D full-resolution surface reconstruction of the above SIM images in respective channels or with GFP-Centrin respectively. Scale bar=5 µm.

B) U-2OS cells were treated with either control, MLL#1 or WDR5#1 for 72 hr and microtubule (MT) regrowth assay was performed. MTs were allowed to regrow for 25 mins and cells were stained with α-tubulin (green). γ-tubulin(red) marks the centrosome. Scale bar =10 µm.

C-D) Cells were scored for the centrosomal and global- MT nucleation after treatment with Control, MLL#1(**C**), or WDR5#1(**D**) siRNA as shown in **B**. Percentages of cells are plotted as shown. (*n*=160 centrosomes, *m*=2 experiments). Error bars represent SEM. ****$P \leq 0.0001$, ns: not significant (two-way ANOVA with Sidak's multiple comparison)

E) Schematic representation of FLAG-MLL constructs used in the study. FLAG-MLLΔTAD and FLAG-MLLΔSET were created by deleting the respective domains while in the case of FLAG-MLLΔSETΔWin, a point mutation was created to mutate the WIN (WDR5 interacting) motif in addition to the deletion of SET domain. MLL^{C3247T} is the predictive translation of the endogenous protein in the case of WSS patient samples 60852 and 60853. The protein gets a premature stop codon at p. A1083*. F-MLL (full length) denotes FLAG epitope-tagged MLL protein with all its domains intact whereas F-MLLΔSET and ΔTAD denote recombinant MLL protein devoid of its SET or transactivation domain respectively. Domains of MLL which impart its chromatin binding capabilities are shown as indicated: AT hooks bind to minor grove of DNA, Zinc finger (Zn) CXXC domain helps in recognition of unmethylated CpG islands,

Bromodomains (BROMO) are essential for protein-protein interactions, plant homeodomain (PHD) of MLL help it to read histone marks. The 'FY' rich N-terminal (FYRN) and 'FY' rich C-terminal (FYRC) domains are required for the hetrodimerization of MLL_N with MLL_C subunits. WDR5 interacting (WIN) motif is responsible for interacting with WDR5. SET, Su(var)3-9, Enhancer-of-zeste, Trithorax domain, TAD, transcriptional activation domain.

F-G) IFS of U-2OS cells stably expressing FLAG-MLL (**F**) or FLAG-WDR5(**G**) constructs used in the study. (**F**) FLAG-MLL, FLAG-MLLΔTAD, FLAG-MLLΔSET and FLAG - MLLΔSETΔWin or (**G**) FLAG-WDR5 and FLAG-WDR5 F133L were labelled with antibodies against FLAG epitope (green), PCNT (red) and DNA (blue). The upper panel is a control for FLAG epitope staining in WT U-2OS cells. Scale bar =20 µm (**F**) and 5 µm (**G**) respectively.

H) MT regrowth assay was performed on IMR90-tert cells 72hr after treatment with Control, MLL or WDR5 siRNA. MTs were allowed to regrow for 15 mins and cells were stained with α-tubulin (green), γ-tubulin (red) marks the centrosomes and and DAPI (blue). Inset shows the zoomed region of aster regrowth. Scale bar = 10 µm

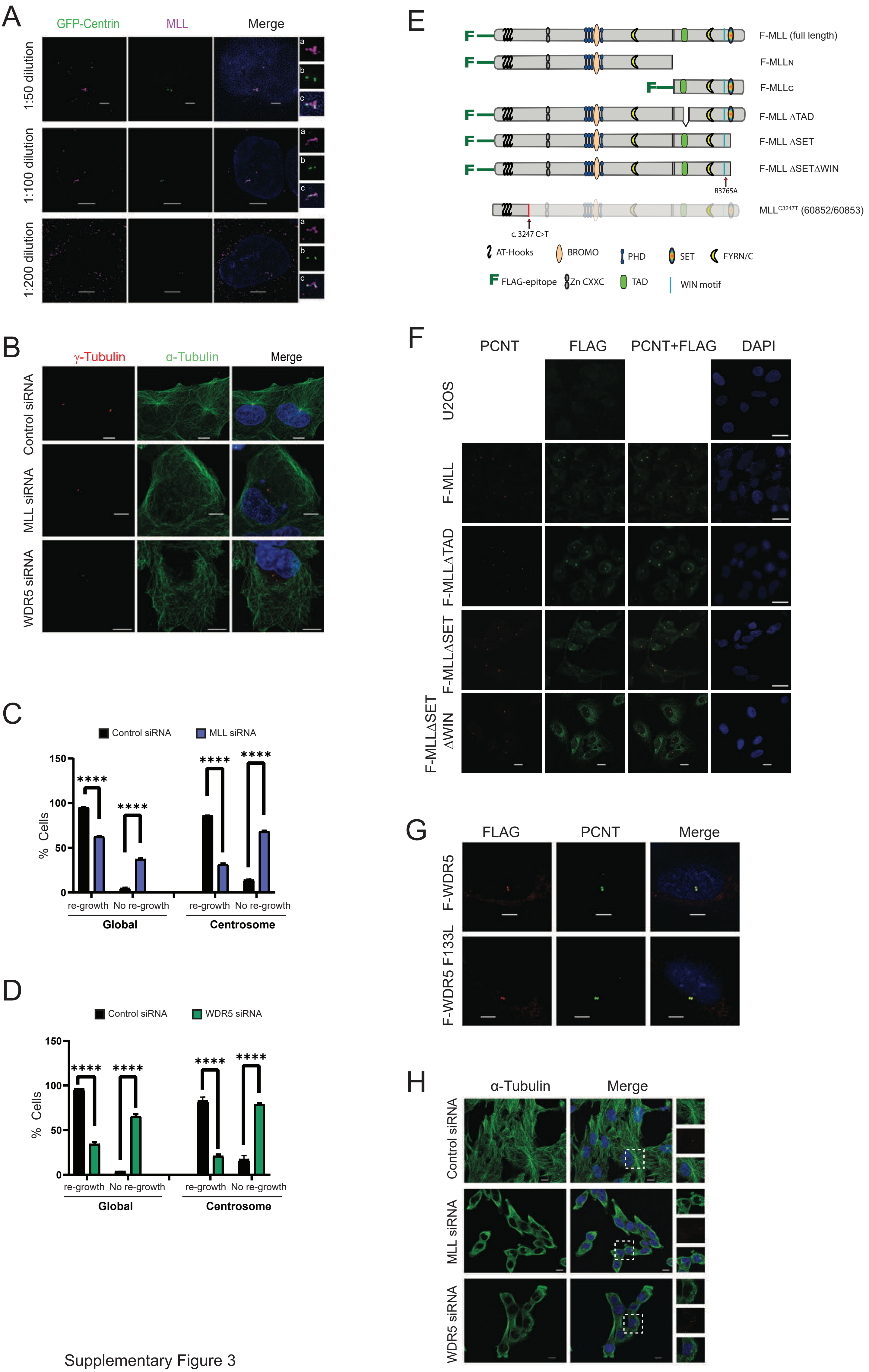

Supplementary Figure 3

Figure S4: MLLc D3 regulates microtubule nucleation and regrowth.

A) Schematic representation of fusion constructs of MLLc used in this study is shown. Green Fluorescence Protein (GFP)-tagged MLLc fragments are shown on the top [GFP-MLLcD1(2718-3275aa), GFP-MLLcD2(3281-3740aa), GFP-MLLcD3(3741-3969aa)] whereas glutathione-S-transferase (GST) tagged fragments of MLLc are shown towards the bottom [GST-MLLcD1(2723-3092aa), GST-MLLcD2a(3084-3445aa), GST-MLLcD2b (3448-3694aa), GST-MLLcD3(3694-3969aa)]. Previously described GST-MLLcD1 and D2 (*19*) were divided into three fragments (D1, D2a and D2b) to ensure stable expression of GST fusion constructs. TAD, transcriptional activation domain; FYRC, "FY-rich" domain C terminus; WIN, WDR5 interacting domain; SET, Su(var)3-9, Enhancer-of-zeste, Trithorax domain; post SET, C-terminal to the SET domain.

B) MT regrowth assay was performed in U-2OS cells stably expressing GFP-MLLc deletions (GFP-MLLcD1, GFP-MLLcD2, GFP-MLLcD3) as described above. Cells were stained with α-tubulin (green) and DAPI (blue). Scale = 10μm. Magnified images are shown inset.

C) U-2OS cells stably expressing GFP-MLL deletions (GFP-MLLcD1, GFP-MLLcD2, GFP-MLLcD3) were scored for aster formation upon endogenous MLL depletion. The percentage of cells was calculated with respect to each individual cell line as well as an averaged control. Both values showed significant results, except for GFP-MLLcD3. Here the plot is shown with averaged control (n=90, m=2 experiments). Error bars represent the SEM. ****$P \leq 0.0001$, ns: not significant (two-way ANNOVA with Tukey's multiple comparison).

D) Localization of GFP-MLL deletions (GFP-MLLcD1, GFP-MLLcD2, GFP-MLLcD3) to the centrosome is shown (in green), with γ-tubulin (red). Scale bar=10μm.

E-F) U-2OS cells stably expressing FLAG-MLL deletions (SFB-MLLcD3 and SFB MLLcD3ΔWIN; R3765A) labelled with α-FLAG (green) and their localization is shown along with the centrosome marker γ-tubulin (red). The nucleus was stained with DAPI. Zoomed

images are shown in the inset. Scale bar= 5μm. SFB, S protein, FLAG, and streptavidin-binding peptide tag. The mean intensity of FLAG staining was quantified and plotted as shown with SEM in **F**. ns: not significant (unpaired student t-test).

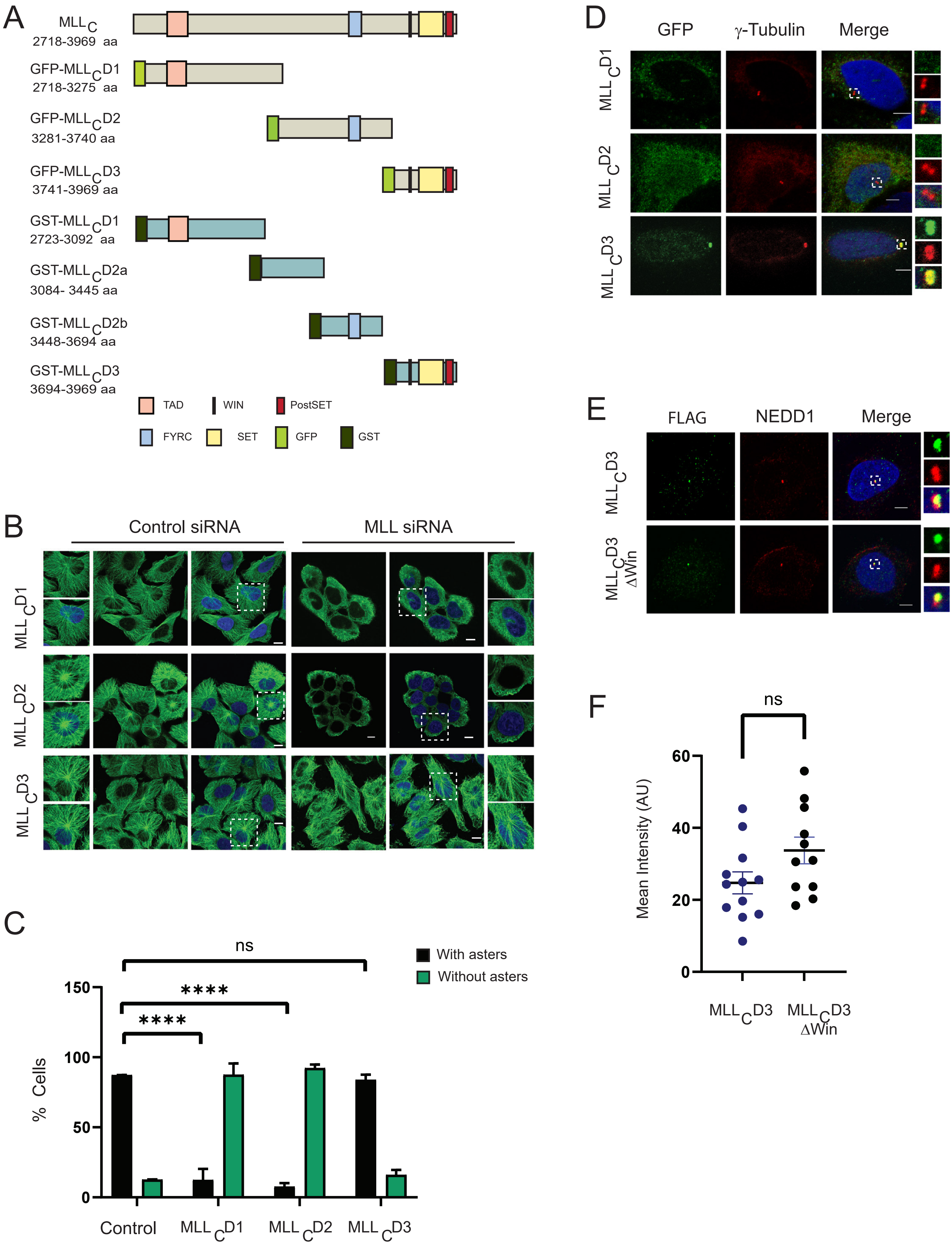

Supplementary Figure 4

Figure S5: The MLL/WDR5 complex interacts with multiple proteins involved in MT nucleation.

A) Immunofluorescence staining (IFS) was performed in U-2OS cells with antibody against KIF2A (green) and γ-tubulin (red) after 72hr treatment with Control or KIF2A siRNA. The nucleus was stained with DAPI (blue) and is shown here in the merged image. Scale bar=5µm. The inset shows the zoomed region of the centrosome of the respective panel.

B) Immunoblot analysis of U-2OS whole cell lysate after treatment with Control or Kif2A siRNA. The blot was probed with α-Kif2A and α-tubulin as shown.

C) MT regrowth assay was performed in U-2OS cells treated with Control or Kif2A siRNA. Regrowth was assessed by α-tubulin (green), and GCP2 (red) staining. Scale bar=5 µm Zoomed inset shows the region marked by the white box.

D) U-2OS cells were quantified for the presence or absence of asters formation after MT regrowth experiment in the Control or Kif2A siRNA-treated cells, and shown as the number of cells (n=100, m=2 experiments). Error bars represent SEM. ns: not significant (two-way ANNOVA with Sidak's multiple comparison test).

E) U-2OS cells stably expressing -Cep72-GFP were stained with α-GFP (green) and NEDD1 (red). The region marked by the white box is zoomed and shown as inset. Scale bar=2µm.

F) IFS was performed in U-2OS cells to visualize endogenous Cep72 (green) and PCNT (red) using specific antibodies after 72hr treatment with Control or Cep72 siRNA. Scale bar=2µm. The inset shows the zoomed region of the centrosome of the respective panel.

G) Whole cell lysate of U-2OS cells was subjected to immunoblotting after 72 hr of siRNA treatment with Control or Cep72 siRNA to analyse the cellular level of protein after the knockdown. The blot was probed with α-Cep72 and α-tubulin as shown.

H-I) Microtubule regrowth assay was performed in U-2OS cells treated with the Control or Cep72 siRNA. The cells were fixed after 15mins of the MT regrowth, stained with α-α-tubulin (green), GCP2 (red) and imaged. Scale bar=5µm. The Zoomed insets show the marked cropped region. **I**) The cells were quantified for the presence and absence of microtubule regrowth and the percentage of cells in each case is plotted (*n*=100, *m*=2 experiments). Error bars represent SEM. ****$P \leq$ 0.0001, ns: not significant (two-way ANNOVA with Sidak's multiple comparison).

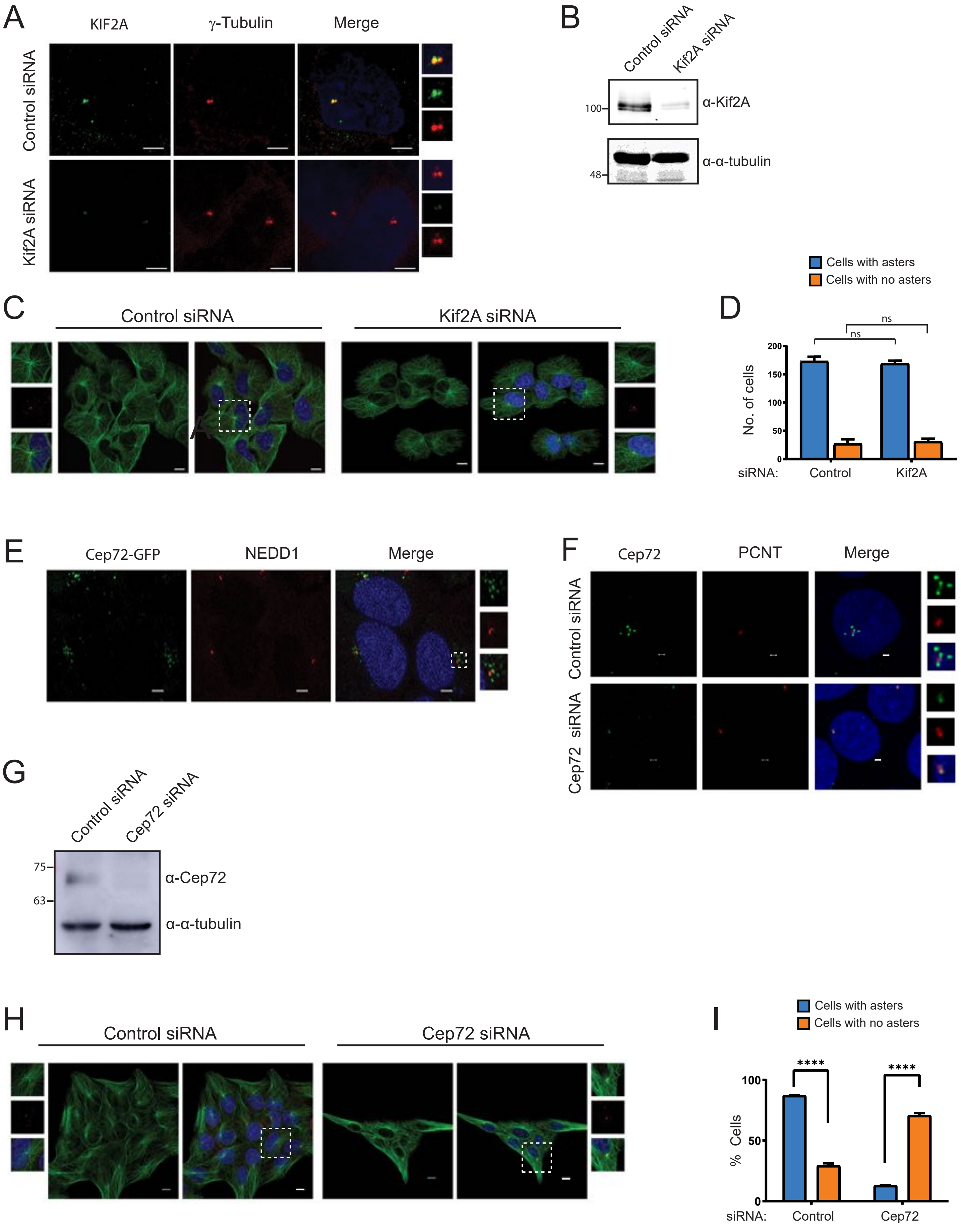

Supplementary Figure 5

Figure S6: Downregulation of MLL/WDR5 affects the localization of centrosomal components during interphase and mitosis.

A) Whole cell lysate of U-2OS cells treated with the Control, WDR5, Cep72 or MLL siRNA cells were analysed for the cellular protein levels of MLL, WDR5, GCP2, γ-tubulin and Cep72. The immunoblots were probed with α-MLL, α-WDR5, α-GCP2, α-γ-tubulin, α-Cep72 antibodies. α-tubulin was used as loading control and is shown below the respective blots. Panel a was detected using ImageQuant LAS500 (chemiluminescence based) whereas b-c were detected with LICOR (fluorescence based) imaging systems.

B) Immunoblot quantifications of MLL, WDR5, Cep72, GCP2 and γ-tubulin levels in Control, WDR5, Cep72 or MLL siRNA-treated cells are shown. Error bars represent the mean with SD. ***P*≤ 0.002, ns: not significant (ordinary one-way ANNOVA Dunnett test).

C-D) U-2OS cells were stained for **C)** NEDD1 (green) or **D)** PCNT (green) and DAPI (blue) in mitosis after 72hr treatment with Control, MLL, WDR5 or Cep72 siRNA. Scale bar= 5µm. The inset shows the zoomed-in single centrosome, marked in the white box. Centrosome levels of these proteins were quantified using Zen software and the mean intensities were plotted. Error bars represent SEM. *****P*≤ 0.0001 (One-way ANNOVA with Bonferroni's multiple comparison test). Scale bar=5µm. AU, arbitrary units.

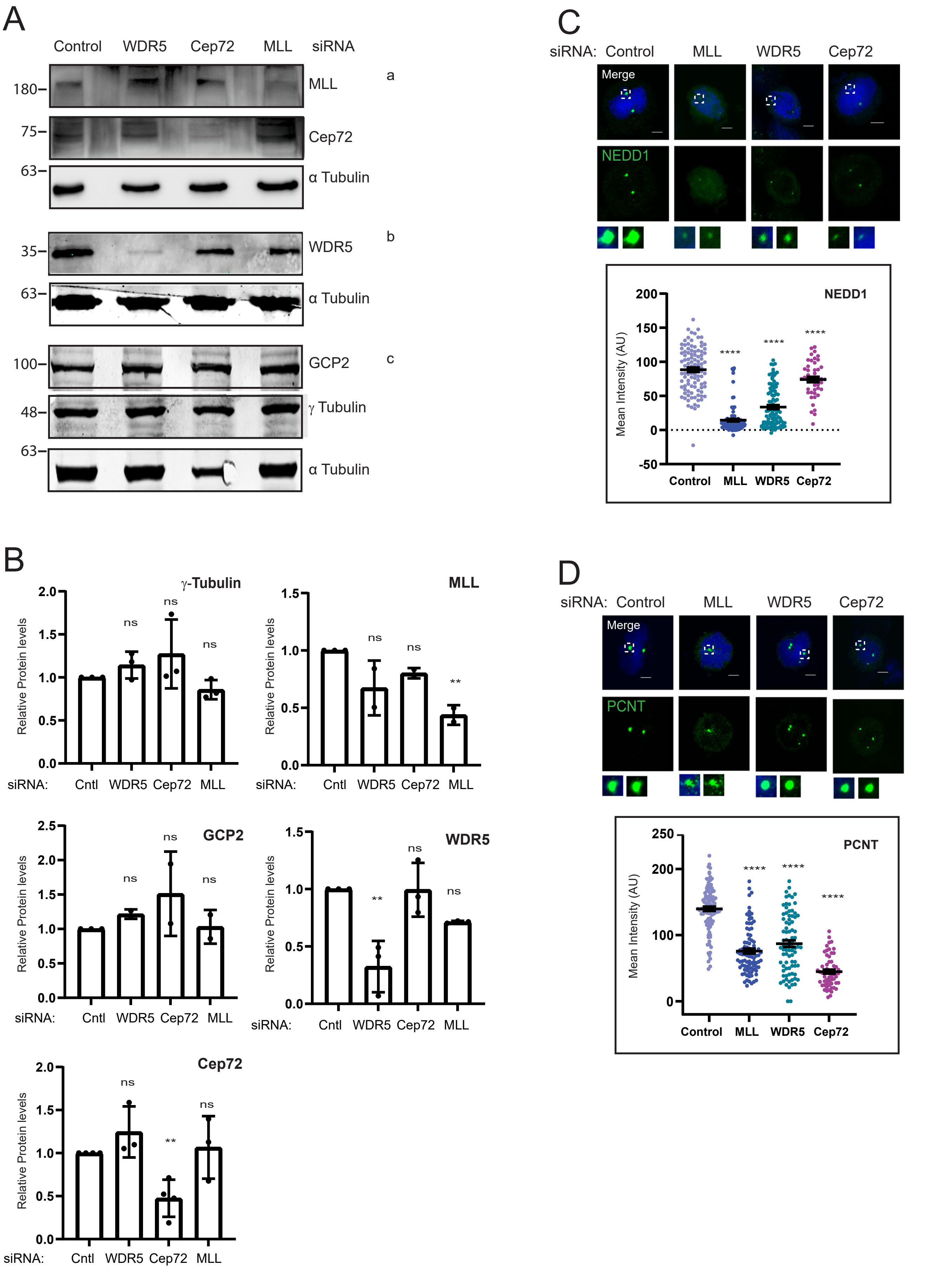

Supplementary Figure 6

Figure-S7: Loss of MLL/WDR5 affects MT-nucleation and spindle formation during mitosis.

A) U-2OS cells stably expressing GFP-tagged Tubulin and mCherry-tagged Histone 2B (H2B) were imaged live at 40X magnification after treating them with the Control, MLL or WDR5 siRNA for 72hr Representative time-lapse images of the mitotic spindle are shown in grey or green (tubulin) and DNA is shown in red (H2B-mCherry). The entire series is available as Movie S5-7. Time is in HH:MM format. Scale bar=5µm.

B) Distribution of exogenously over-expressed Cep72-GFP protein in the Control, MLL or WDR5 siRNA treated cells is shown. Merge and separate channels for GFP fluorescence (green), anti-γ-tubulin antibody (red) and DAPI staining are shown. As γ-tubulin levels were reduced after MLL and WDR5 siRNA treatment, we have artificially enhanced the signal in merged image (and inset of the same) to mark the centrosome clearly. Scale bar=5µm.

C) Centrosomal levels of Cep72-GFP and γ-tubulin proteins were quantified from the Control, MLL or WDR5 siRNA-treated cells and plotted (*n*=100, *m*=2 experiments). Error bars represent SEM., *****P*≤ 0.0001, ****P*≤ 0.0008 (One-way ANNOVA with Tukey's multiple comparison test). AU, arbitrary units.

D) Immunofluorescence was performed on U-2OS cells stably expressing MLLcD3 and MLLcD3ΔWin (R3765A) after treating with the Control or MLL#2 siRNA for 72 hr Merge and separate channels for Cep72 (green), PCNT (red) and DAPI are shown. Scale bar=5µm.

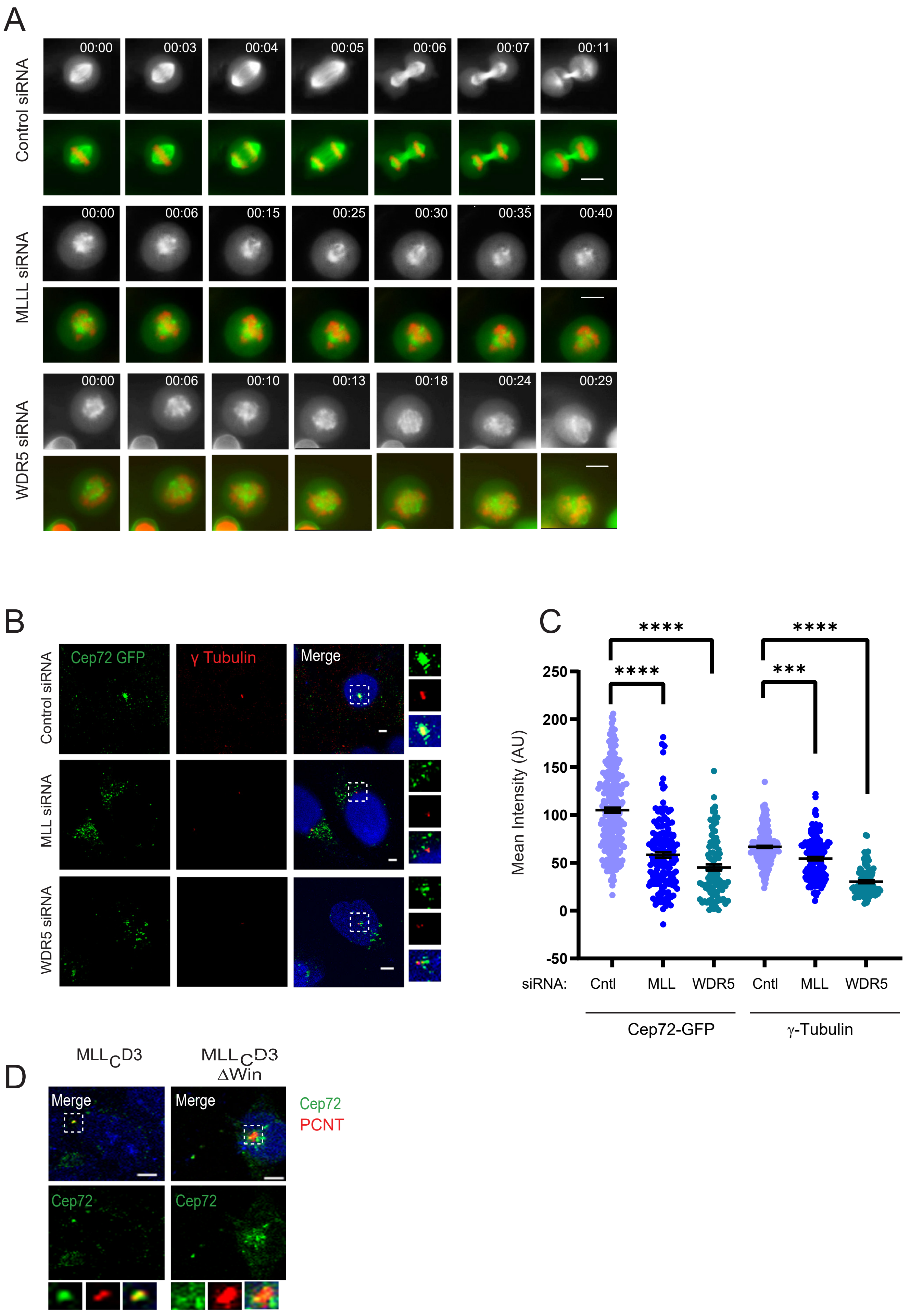

Supplementary Figure 7

Figure S8: MT-nucleation and Cep72 recruitment are affected in WSS patient-derived cells.

A-B) IFS of lymphoblastoid cells of the Control and WSS patients to show the centrosomal staining of **A)** GCP2 (green) and AKAP9 (red), **B)** CDK5RAP2 (green) and γ-tubulin (red). The nucleus was stained with DAPI and shown in blue in the merged images. Scale bar=2μm.

C) Centrosomal staining of NEED1, PCNT and CDK5RAP2 in lymphoblastoid **(**Control, 60852, 60853) cells were quantified and shown (*n*=400, *m*=4 experiments). Error bars represent SEM. ****$P\leq$ 0.0001, ns: not significant (One-way ANNOVA with Tukey's multiple comparisons). AU , arbitrary units.

D) Cellular protein levels of MLL, WDR5, CEP72, GCP2, γ-tubulin**,** NEDD1 analysed in the whole cell lysate of WSS patients lymphoblastoid cell lines (60852, 60853) and the two healthy individuals (control1, control2). α-tubulin blot as the loading control for each blot is shown below. Each protein lane is normalized to the tubulin and fold change is compared with the control1 as shown. MLL blot is the same as shown in Figure 8A.

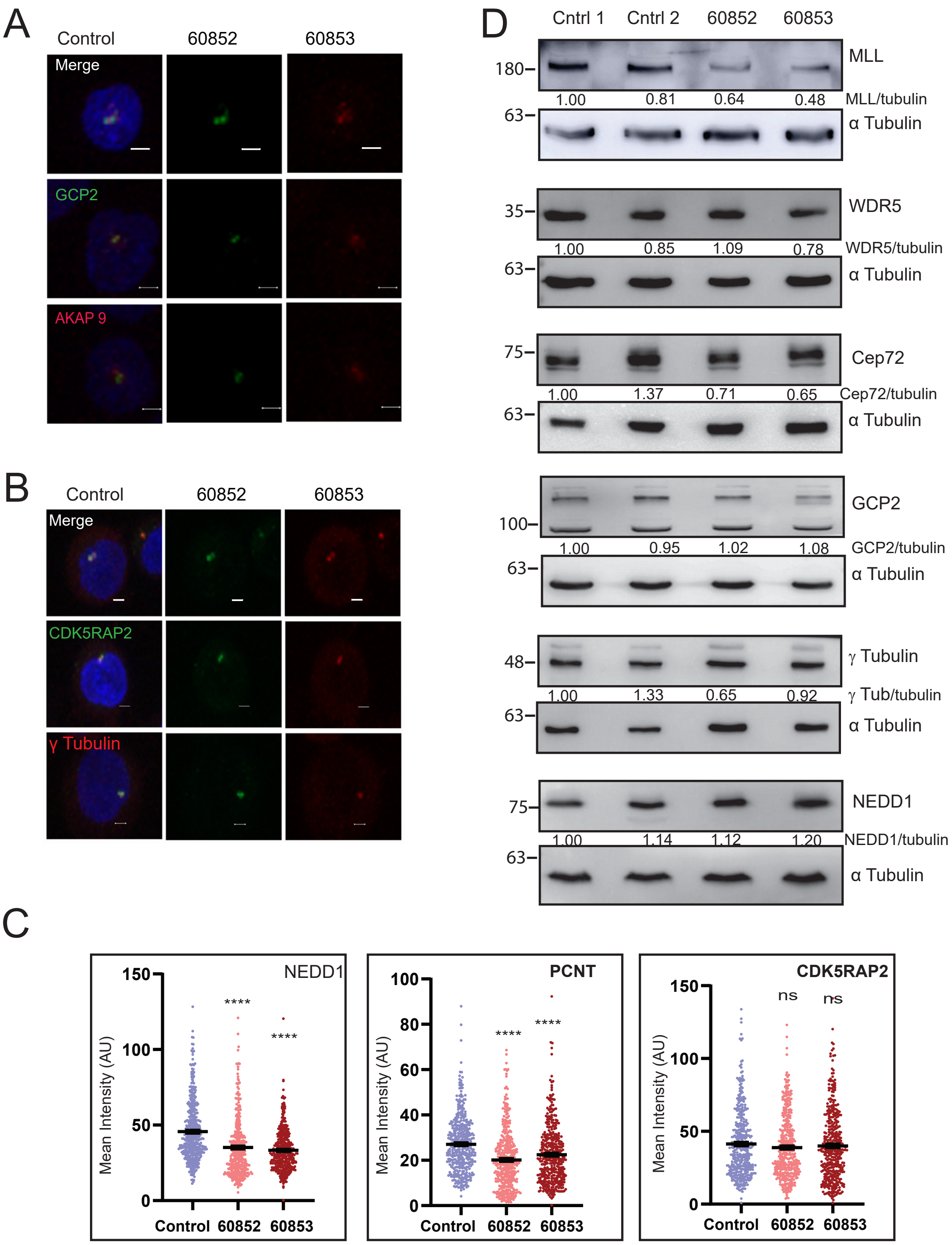

Supplementary Figure 8

Table S1: Raw data : All the underlying data used in this study is provided here.

Supplementary Movie Legends

Movie S1-4. Time-lapse images of GFP-Tubulin, H2B-mCherry expressing U2OS cell in mitosis after depolymerizing microtubules. Related to Figure 5.

U-2OS cells stably expressing H2B-mCherry and GFP-Tubulin were treated with the Control (movie S1), MLL (movie S2), or WDR5 (movie S3), or Cep72 (movie S4), siRNA. Time-lapse images were taken at an interval of 1 min after depolymerizing MT and are displayed at 1 frame per second for the Control siRNA, MLL siRNA, WDR5 siRNA treated cells. The length of the movie for the Control siRNA and MLL siRNA is 11 min, for the WDR5 siRNA is 10 min, for the Cep72 siRNA is 12 min. Scale = 5µm.

Movie S5-7. Time-lapse images of GFP-Tubulin, H2B-mCherry expressing U2OS cell in mitosis. Related to Figure S4.

U-2OS cells stably expressing H2B-mCherry and GFP-Tubulin were treated with Control (movie S5), MLL (movie S6) or WDR5 (movie S7) siRNA. Time-lapse images were captured at an interval of 1 min and are displayed at 3 frames per second for the Control and 5 frames per second for MLL. WDR5 siRNA movies were captured at an interval of 2 min and are displayed at 5 frames per second. The length of the movie for the Control siRNA is 11 mins, 41 mins for MLL siRNA and 47 mins for WDR5 siRNA. The movie is deconvoluted using AutoquantX3 and analyzed using NIS elements and FIJI software. Scale = 5µm.

Figure S9: Blot Transparency: Original uncropped blots used in this study.

Figure S9: Blot Transparency: Original uncropped blots used in this study are provided here.

Figure S1

B

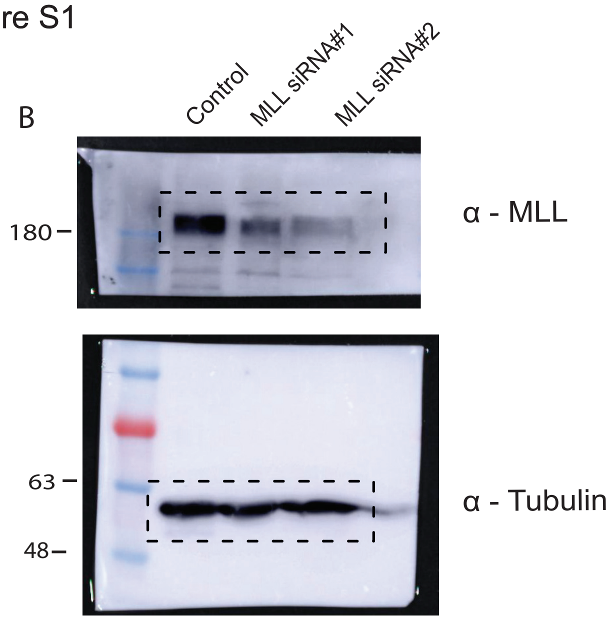

C

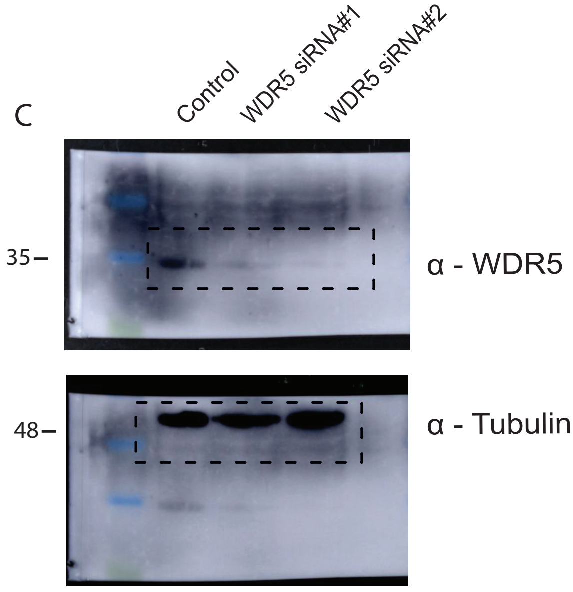

Figure S5

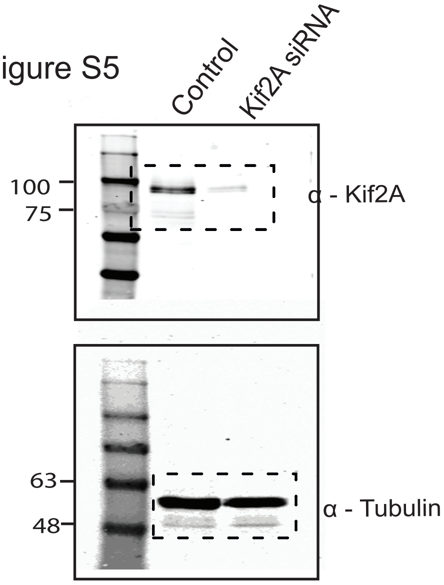

G

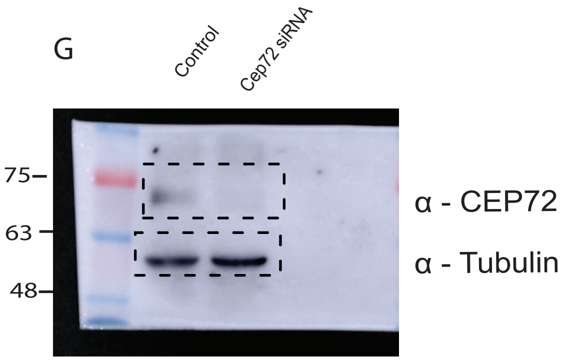

Figure S6

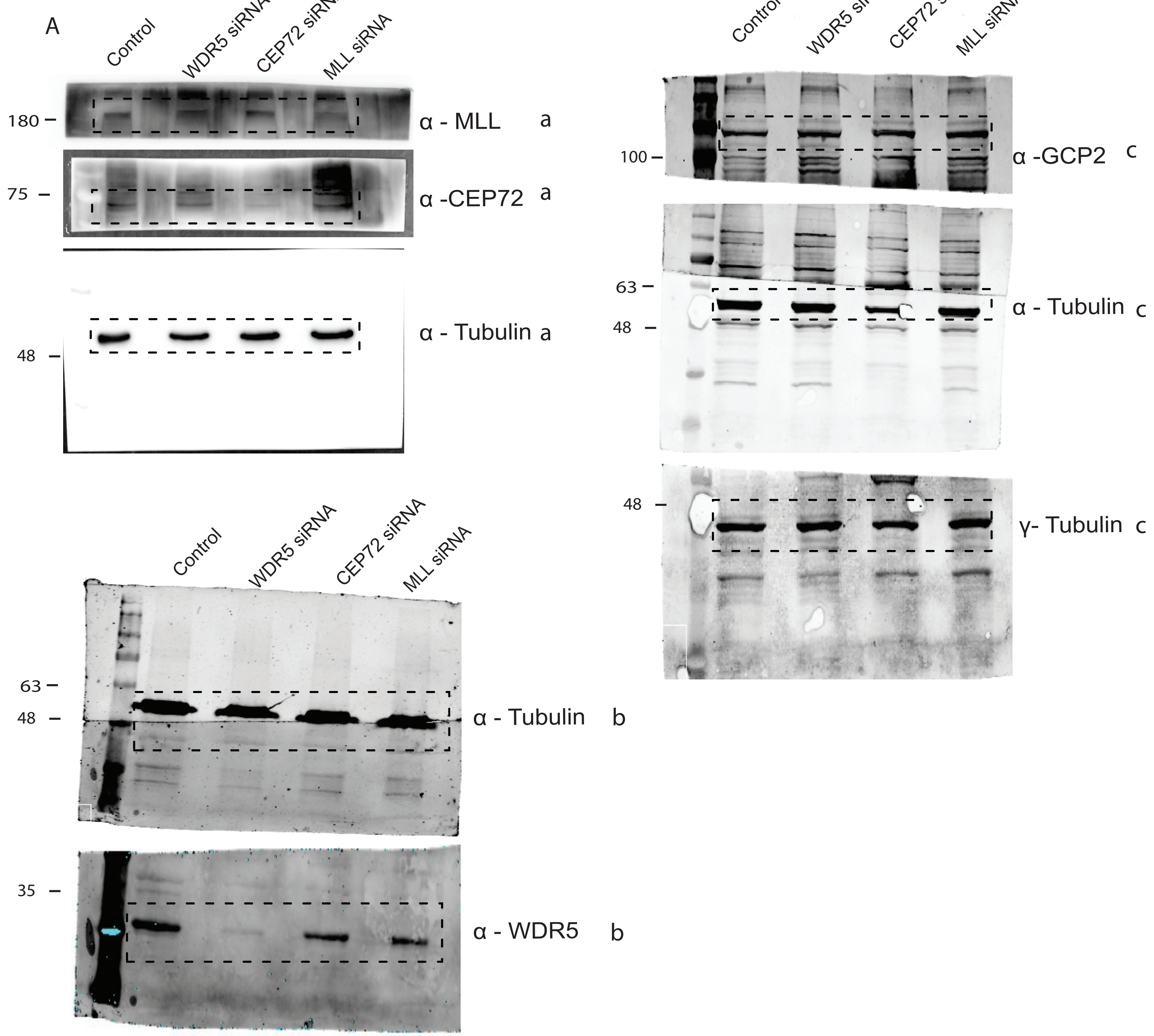

A
Control
WDR5 siRNA
CEP72 siRNA
MLL siRNA
180
α - MLL a
75
α -CEP72 a
48
α - Tubulin a
100
α -GCP2 c
63
48
α - Tubulin c
48
γ- Tubulin c
63
48
α - Tubulin b
35
α - WDR5 b

Figure S8

F

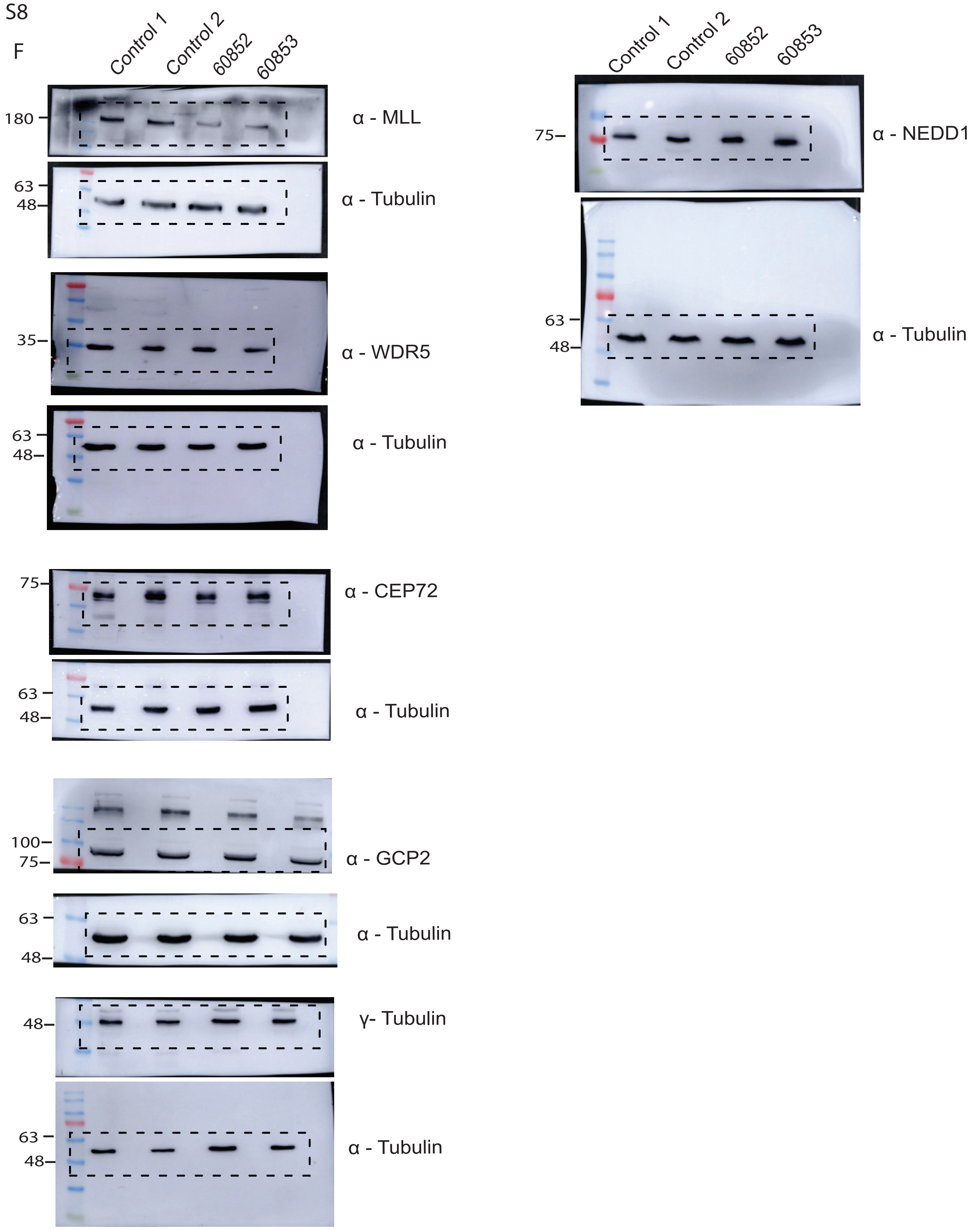
Control 1
Control 2
60852
60853
180
α - MLL
63
48
α - Tubulin
35
α - WDR5
63
48
α - Tubulin
75
α - CEP72
63
48
α - Tubulin
100
75
α - GCP2
63
48
α - Tubulin
48
γ- Tubulin
63
48
α - Tubulin
Control 1
Control 2
60852
60853
75
α - NEDD1
63
48
α - Tubulin
